# S19W, T27W, and N330Y mutations in ACE2 enhance SARS-CoV-2 S-RBD binding toward both wild-type and antibody-resistant viruses and its molecular basis

**DOI:** 10.1038/s41392-021-00756-4

**Published:** 2021-09-16

**Authors:** Fei Ye, Xi Lin, Zimin Chen, Fanli Yang, Sheng Lin, Jing Yang, Hua Chen, Honglu Sun, Lingling Wang, Ao Wen, Xindan Zhang, Yushan Dai, Yu Cao, Jingyun Yang, Guobo Shen, Li Yang, Jiong Li, Zhenling Wang, Wei Wang, Xiawei Wei, Guangwen Lu

**Affiliations:** 1 West China Hospital Emergency Department (WCHED), State Key Laboratory of Biotherapy, West China Hospital, Sichuan University, Chengdu, Sichuan China; 2 Disaster Medicine Center, West China Hospital, Sichuan University, Chengdu, Sichuan China; 3 Laboratory of Aging Research and Cancer Drug Target, State Key Laboratory of Biotherapy and Cancer Center, National Clinical Research Center for Geriatrics, West China Hospital, Sichuan University, Chengdu, Sichuan China

**Keywords:** Molecular biology, Structural biology

## Abstract

SARS-CoV-2 recognizes, via its spike receptor-binding domain (S-RBD), human angiotensin-converting enzyme 2 (ACE2) to initiate infection. Ecto-domain protein of ACE2 can therefore function as a decoy. Here we show that mutations of S19W, T27W, and N330Y in ACE2 could individually enhance SARS-CoV-2 S-RBD binding. Y330 could be synergistically combined with either W19 or W27, whereas W19 and W27 are mutually unbeneficial. The structures of SARS-CoV-2 S-RBD bound to the ACE2 mutants reveal that the enhanced binding is mainly contributed by the van der Waals interactions mediated by the aromatic side-chains from W19, W27, and Y330. While Y330 and W19/W27 are distantly located and devoid of any steric interference, W19 and W27 are shown to orient their side-chains toward each other and to cause steric conflicts, explaining their incompatibility. Finally, using pseudotyped SARS-CoV-2 viruses, we demonstrate that these residue substitutions are associated with dramatically improved entry-inhibition efficacy toward both wild-type and antibody-resistant viruses. Taken together, our biochemical and structural data have delineated the basis for the elevated S-RBD binding associated with S19W, T27W, and N330Y mutations in ACE2, paving the way for potential application of these mutants in clinical treatment of COVID-19.

## Introduction

In the end of 2019, a novel coronavirus, designated as severe acute respiratory syndrome coronavirus 2 (SARS-CoV-2), suddenly emerged and swiftly spread globally.^1–3^ Despite of global efforts to control the viral infection, the pandemic is still surging. The severe threat of SARS-CoV-2 to the global public health makes it an urgent issue to develop effective anti-viral therapeutics.

SARS-CoV-2 is an enveloped virus with a single-stranded positive-sense RNA genome of about 30 kb in size.^2–4^ The virus encodes four structural proteins, including spike (S), envelope (E), membrane (M), and nucleoprotein (N).^2,3,5^ Of these viral proteins, the surface-located and envelope-interspersed S protein plays a key role in the viral pathogenesis by mediating virus entry. It can, on one hand, recognize the cellular receptor/(s) to tether virus particles onto the surface of susceptible host cells and on the other promote the fusion between viral envelop and host cell membrane to release the viral genome and set up infection.^2,3,5,6^ Therefore, reagents that can disrupt the S/receptor interaction, as exemplified by the sera from the recovered patients^7–9^ and a series of identified neutralizing monoclonal antibodies, represent promising anti-viral therapeutics for the treatment of COVID-19.^6,10–12^

Previous studies have shown that the human angiotensin-converting enzyme 2 (ACE2) is a functional cellular receptor for SARS-CoV-2.^2,13^ ACE2 is a type I trans-membrane protein with a large N-terminal ecto-domain, which can be further divided into a peptidase domain (PD) possessing the carboxypeptidase activity and a collectrin-like domain (CLD) mediating ACE2 homodimerization.^14,15^ During virus infection, however, ACE2 has been shown to be exploited by multiple coronaviruses, including SARS-CoV, SARS-CoV-2, and human coronavirus NL63, for cell entry.^2,13,16–18^

Several structural and functional studies have shown that the specific binding of SARS-CoV-2 to ACE2 is mediated by the receptor-binding domain of the viral S protein (S-RBD) directly engaging the PD domain of the receptor.^15,19–21^ The previously reported complex structures of SARS-CoV-2 S-RBD bound to ACE2 demonstrate that the new coronavirus shares a similar receptor-binding mode to that of SARS-CoV.^15,20,21^ Nevertheless, real-time binding data reveal a much higher ACE2-binding affinity for SARS-CoV-2 S-RBD than SARS-CoV S-RBD.^20–23^ Recombinant ACE2 has been shown to block SARS-CoV-2 entry in vitro^24–26^ and exhibit promising therapeutic efficacy in vivo.^27^ In addition, recent studies further show that the decoy ACE2 protein could be optimized to enhance its S-RBD binding capacity and subsequently to increase its efficacy in inhibiting SARS-CoV-2 infection.^28,29^

In this study, we found that individual substitutions of ACE2 residues S19, T27, and N330 with W19, W27, and Y330 could moderately enhance S-RBD engagement. In addition, Y330 could be synergistically combined with either W19 or W27 to further increase the S-RBD binding affinity, but W19 and W27 were mutually incompatible. In order to learn the basis underlying such residue preference at these positions, we further solved the crystal structures of SARS-CoV-2 S-RBD bound to these affinity-enhanced ACE2 mutants. Our structures unexpectedly showed an ACE2 molecule trapped in a closed conformation in complex with the viral ligand. Structural analyses revealed that Y330 and W19/W27 were distantly located along the interface, devoid of any steric interference, and therefore mutually beneficial. Residues W19 and W27, however, were observed to orient their side-chains toward each other and would otherwise cause steric conflicts, correlating well with their incompatibility. In addition, we also showed that these residue substitutions also dramatically enhanced the ACE2 binding to SARS-CoV S-RBD and that such enhanced binding correlated with increased inhibitory efficacy in blocking virus entry toward both wild-type and antibody-resistant SARS-CoV-2.

## Results

### Identification of key interface residues in ACE2 toward enhanced SARS-CoV-2 spike binding

**Fig. 1:**
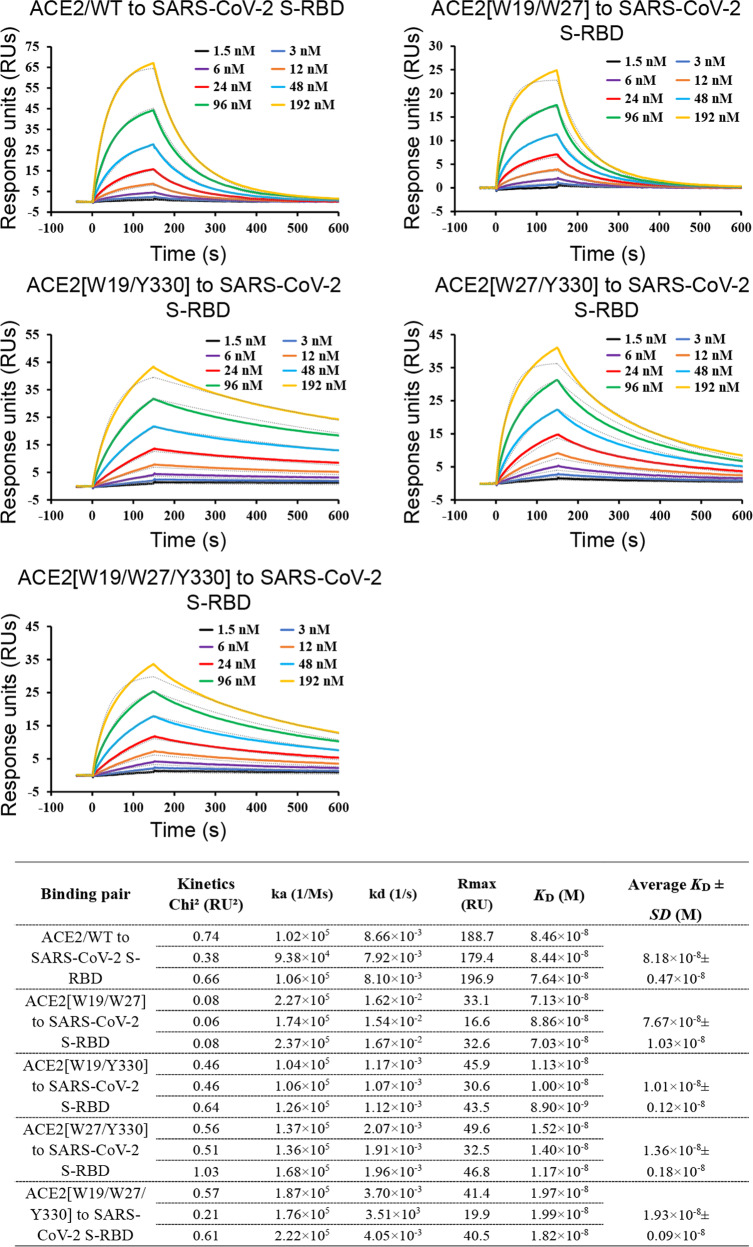
An SPR assay characterizing the real-time binding kinetics of the ACE2 proteins (wild-type and mutants) to SARS-CoV-2 S-RBD. Three independent experiments are conducted and the recorded profiles from one representative experiment are shown. The slow-on/slow-off kinetic data are analyzed by the 1:1 binding model. The calculated kinetic parameters are summarized. ACE2/WT: wild-type ACE2; ACE2[W19/W27]: ACE2 containing the S19W, T27W, H374A, H378A, and E402A mutations; ACE2[W19/Y330]: ACE2 containing the S19W, N330Y, H374A, H378A, and E402A mutations; ACE2[W27/Y330]: ACE2 containing the T27W, N330Y, H374A, H378A, and E402A mutations; ACE2[W19/W27/Y330]: ACE2 containing the S19W, T27W, N330Y, H374A, H378A, and E402A mutations

In light of the potential application of soluble ACE2 in clinical treatment of COVID-19,^27,30^ our initial goal was to identify residue-positions in ACE2 at which amino acid substitutions could result in enhanced binding to SARS-CoV-2 S-RBD. We first compared a series of reported SARS-CoV-2 S-RBD/ACE2 complex structures (PDB codes: 6LZG, 6M0J, 7C8D, 7DDO, and 7DDP) by superimposition, which expectedly revealed highly conserved binding interface between the S-RBD ligand and the ACE2 receptor (Supplementary Fig. 1a). This ligand/receptor-binding interface was therefore selected for computer-aided virtual screening using MOE (Molecular Operating Environment 2019.0102 from the Chemical Computing Group). A series of mutation options in ACE2 were subsequently revealed, which included S19W, T27W, H34E, G326W, G326K, N330Y, and K353R (Supplementary Fig. 1b). The PD-domain proteins of these mutants, as well as those of the wild-type (WT) and the peptidase active-site mutated (triple mutations with H374A, H378A, and E402A, and designated as active-site-mutant) ACE2s, were subsequently prepared from insect cells (Supplementary Fig. 2) and tested for their binding affinities to SARS-CoV-2 S-RBD by surface plasmon resonance (SPR). As expected, ACE2/WT readily interacted with S-RBD and showed a dissociation constant (*K*_D_) of 81.8 ± 4.7 nM (Fig. 1), coinciding well with previous reports when SARS-CoV-2 S-RBD was immobilized on the chips and ACE2 was used as the analyst.^20^ Of the mutants tested, ACE2/S19W, ACE2/T27W, and ACE2/N330Y were shown to exhibit moderately elevated binding, showing an affinity of 48.6 ± 12.8 nM, 38.5 ± 8.5 nM, and 29.3 ± 10.4 nM, respectively. In addition, ACE2/active-site-mutant, with an affinity of 74.5 ± 13.9 nM, also showed a slightly enhanced binding. The remaining mutants, however, were of apparently lower binding affinities than the WT protein (decreased binding for ACE2/H34E, ACE2/G326K, and ACE2/K353R and no observable binding for ACE2/G326W) (Supplementary Fig. 3).

Guided by the results of our initial SPR screening, the ACE2 mutations of S19W, T27W, and N330Y were further combined in pairs or simultaneously in the context of ACE2/active-site-mutant (H374A, H378A, and E402A), which was observed to also slightly benefit SARS-CoV-2 S-RBD binding. The resultant mutant proteins (designated as ACE2[W19/W27], [W19/Y330], [W27/Y330], and [W19/W27/Y330] hereafter, Supplementary Fig. 2) were then subjected to SPR binding assay in parallel with the WT protein. The determined affinities were 10.1 ± 1.2 nM, 13.6 ± 1.8 nM, 76.7 ± 10.3 nM, and 19.3 ± 0.9 nM for ACE2[W19/Y330], ACE2[W27/Y330], ACE2[W19/W27], and ACE2[W19/W27/Y330], respectively (Fig. 1). The results, therefore, clearly showed a synergistic effect in binding enhancement when Y330 was combined with either W19 or W27 but the mutual incompatibility between W19 and W27.

### Structures of SARS-CoV-2 S-RBD bound to ACE2[W19/Y330] and ACE2[W27/Y330] trapped in a closed conformation

**Fig. 2:**
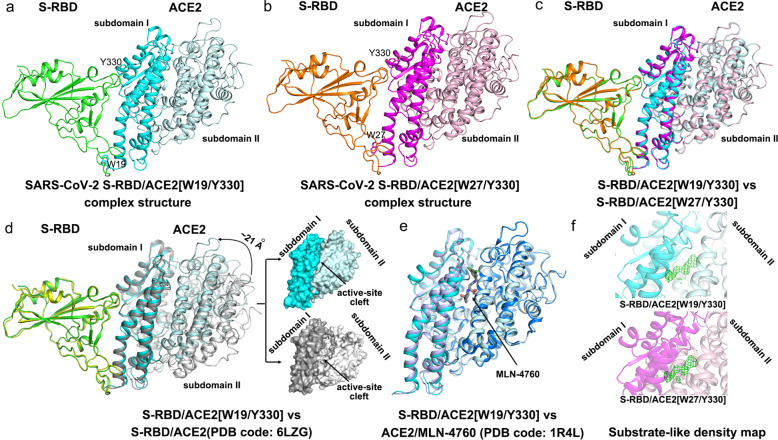
SARS-CoV-2 S-RBD bound to ACE2 trapped in the closed conformation. **a** Crystal structure of SARS-CoV-2 S-RBD in complex with ACE2[W19/Y330]. S-RBD is in green and ACE2 subdomains I and II are colored cyan and pale cyan, respectively. W19 and Y330 are highlighted by sticks. **b** Crystal structure of SARS-CoV-2 S-RBD in complex with ACE2[W27/Y330]. S-RBD is in orange and ACE2 subdomains I and II are colored magenta and light pink, respectively. W27 and Y330 are highlighted by sticks. **c** Superimposition of the two structures presented in (**a**) and (**b**) to highlight the well-aligned S-RBD and ACE2 molecules. **d** Superimposition of our S-RBD/ACE2[W19/Y330] structure (green for S-RBD, cyan for ACE2 subdomain I, and pale cyan for ACE2 subdomain II) onto a previously reported structure of SARS-CoV-2 S-RBD (yellow) bound to wild-type ACE2 (dark gray for subdomain I and light gray for subdomain II) (PDB code: 6LZG). The movement of ACE2 subdomain I toward subdomain II (by about ~21 Å at the outer edge point) in our structure is highlighted. The subsequent change of the active-site cleft in the two structures is shown in a space-filling mode on the right. **e** Superimposition of our S-RBD/ACE2[W19/Y330] structure (cyan for subdomain I and pale cyan for subdomain II) onto a previously reported structure of ACE2 (light blue for subdomain I and marine for subdomain II) in complex with the MLN-4760 inhibitor (PDB code: 1R4L). **f** Dense electron densities for a possible substrate-like peptide observed in the ACE2 active-site cleft in our structures. The electron densities are contoured at 1.5*σ* using the |Fo|–|Fc| map

In order to investigate the basis for the observed binding enhancement, we further solved the crystal structures of SARS-CoV-2 S-RBD bound to ACE2[W19/Y330] and ACE2[W27/Y330], of which the mutation-combinations brought about the best improvement in the binding. The two structures were solved to 2.7 and 2.5 Å and refined to *R*_work_ = 0.1838/*R*_free_ = 0.2242 and *R*_work_ = 0.1903/*R*_free_ = 0.2289, respectively (Supplementary Table 1). Each structure contained a single S-RBD/ACE2 complex bound in a 1:1 binding mode and in each case the viral ligand directly engaged subdomain I of the peptidase receptor (Fig. 2a–c).

Overall, the binding mode observed in our structures was essentially the same to that observed in the previously reported complex structure of SARS-CoV-2 S-RBD bound to ACE2 (PDB code: 6LZG) (Fig. 2d). Comparison of these structures expectedly revealed well-aligned S-RBD molecule and largely-superimposed ACE2 subdomain I (Fig. 2d). The subdomain II of the receptor, however, unexpectedly showed a large conformational difference. The outer edge of the subdomain II in our structures moved as much as ~21 Å toward subdomain I (Fig. 2d), significantly narrowing down the active-site cleft formed between the two subdomains. Such variance reminded us of an ACE2 molecule in a closed conformation when inhibitors bound or during catalysis. We therefore further compared our structures with a previously reported structure of ACE2 bound with MLN-4760 (PDB code: 1R4L), a potent inhibitor for human ACE2.^14^ As expected, the structural superimposition revealed both well-aligned subdomain I and II (Fig. 2e), demonstrating that the ACE2 molecule in our structures was indeed trapped in a closed conformation. We also noticed that a stretch of dense and continuous electron densities, which was large enough in size to accommodate a peptide of 4–5 residues, could be clearly observed in the active-site cleft of ACE2 in the ∣Fo∣-∣Fc∣map (Fig. 2f). In comparison to the ACE2/MLN-4760 complex structure, the stretch of the electron densities apparently overlapped with the MLN-4760 inhibitor (Supplementary Fig. 4). We therefore believe that such electron densities present in the active-site cleft were likely the reason restricting ACE2 in a closed conformation in our structures (Supplementary Fig. 4). Because ACE2[W19/Y330] and ACE2[W27/Y330] also contained the active-site residue mutations (H374A, H378A, and E402A) and were therefore inert in catalysis, we postulated that a substrate-like peptide was accidentally captured in the cleft during expression, locking the receptor in the closed conformation.

### Basis for the aromatic residue preference at ACE2 positions 19, 27, and 330 toward enhanced SARS-CoV-2 S-RBD binding

**Fig. 3:**
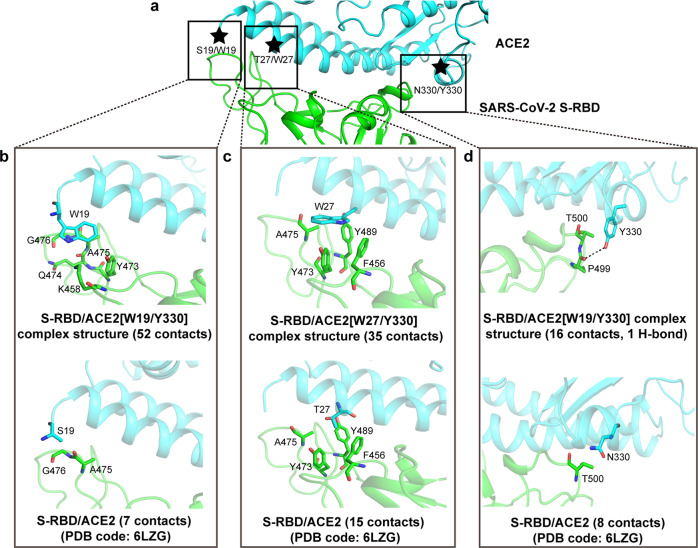
The superior contributions of the S19W, T27W, and N330Y mutations in ACE2 to SARS-CoV-2 S-RBD binding. **a** An overview of the binding interface between ACE2 (in cyan) and SARS-CoV-2 S-RBD (in green) to highlight the steric positions of the S19/W19, T27/W27, and N330/Y330 residues, which are marked with stars. The detailed inter-molecule contacts contributed by these three pairs of amino acids are further delineated in (**b**), (**c**), and (**d**), respectively. The residues referred to in the text are shown and labeled. Dashed line indicates H-bond. **b** Comparison of the inter-molecule interactions mediated by W19 and S19. **c** Comparison of the inter-molecule interactions mediated by W27 and T27. **d** Comparison of the inter-molecule interactions mediated by Y330 and N330. The number of van der Waals contacts is listed in parentheses

Facilitated by our solved structures, we further characterized the detailed amino acid interactions between SARS-CoV-2 S-RBD and the ACE2 mutants, mainly focusing on the interactions associated with the S19W, T27W, and N330Y mutations. Overall, the aromatic-residue substitutions did not induce obvious conformational changes to the neighboring interface residues (4 in the proximity to S19, 8 in the proximity to T27, and 9 in the proximity to N330) in ACE2 (Supplementary Fig. 5a–c), demonstrating that the large side-chains incorporated at these three positions could be individually ‘well-tolerated’. The enhanced binding associated with the S19W, T27W, and N330Y substitutions were, therefore, almost completely contributed by the van der Waals (vdw) interactions mediated by the aromatic side-chains. For W19, the residue was observed to interact with K458, Y473, Q474, A475, G476 in S-RBD and to provide a total of 52 vdw contacts, outnumbering S19 by about sevenfold, which only engaged A475, G476 with 7 vdw contacts (Fig. 3a, b and Supplementary Table 3). For W27, it projected its indole ring into an apolar pocket constituted by residues F456, Y473, A475, Y489 in S-RBD, making 35 vdw contacts for strong hydrophobic interactions. Such engagement significantly outperformed T27, which only made 15 contacts with the apolar pocket (Fig. 3a, c and Supplementary Table 3). For Y330, the amino acid was shown to make 16 vdw contacts and one H-bond with P499 in S-RBD. In contrast, N330 in ACE2/WT only contributed a limited number of inter-molecule contacts via interacting with S-RBD residue T500 (Fig. 3a, d and Supplementary Table 3).

### Structural explanation on mutual incompatibility between W19 and W27

**Fig. 4:**
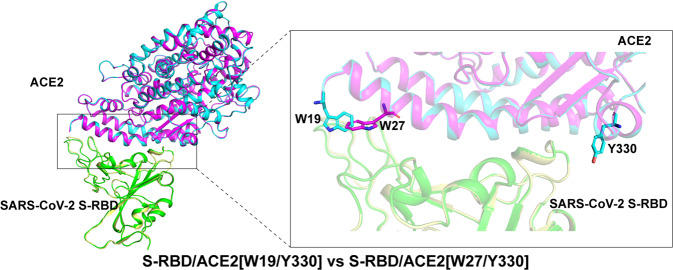
Steric conflicts between W19 and W27 in ACE2 when engaging SARS-CoV-2 S-RBD. The S-RBD/ACE2[W19/Y330] (S-RBD in green and ACE2 in cyan) and S-RBD/ACE2[W27/Y330] (S-RBD in light yellow and ACE2 in magenta) complex structures solved in this study are superimposed onto each other and presented in the left panel. A magnified view of the S-RBD/ACE2-binding interface is highlighted in the right panel. The interface-located residues W19, W27, and Y330 are shown and labeled

It is notable that our SPR binding assays have shown a synergistic effect in binding enhancement when Y330 was combined with either W19 or W27 but the mutual incompatibility between W19 and W27. To gain insight into such residue-combination features, the two structures of S-RBD in complex with ACE2[W19/Y330] and ACE2[W27/Y330] were superimposed onto each other (Fig. 4). Along the binding interface, W19 was located on one terminal side and Y330 on the opposite side. W27, which resided on the first helical component of ACE2, was in the proximity to W19 but was separated from the latter by about two helix-turns. Sterically, Y330 and W19/W27 were distantly located from each other and devoid of any steric interference. W19 and W27, however, were observed to orient their side-chains toward each other, leading to strong steric conflicts. The observations highlighted that, when W19 and W27 were both incorporated into ACE2, the two amino acids would have to adopt alternative conformations that were different from those observed in our structures to engage SARS-CoV-2 S-RBD. Such conflicts in the side-chain orientations between W19 and W27, therefore, correlated well with the mutual incompatibility between the two mutations toward further enhancement in S-RBD binding.

### Improved SARS-CoV-2 entry inhibition toward WT, antibody-resistant, and circulating variant viruses

**Fig. 5:**
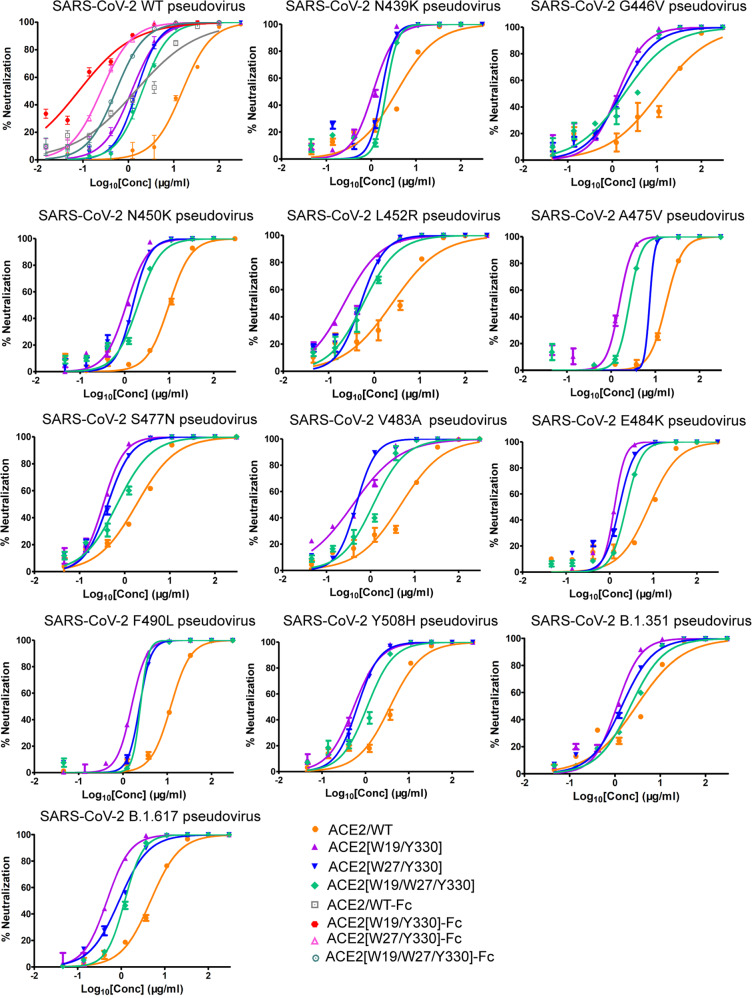
Increased inhibitory efficacy of the indicated ACE2 mutants in blocking virus entry of SARS-CoV-2. The luciferase-incorporated SARS-CoV-2 pseudoviruses (wild-type and antibody-resistant) are pre-incubated with the indicated ACE2 proteins (wild-type and mutants) for 1 h at 37 °C and then used to infect the human ACE2-transfecting 293T cells. Luciferase activities in target are measured. Each point represents the mean ± SD from triplicate experiments

Since ACE2[W19/Y330], ACE2[W27/Y330], and ACE2[W19/W27/Y330] were shown to exhibit the best enhancement in S-RBD binding, we further tested the entry-inhibition efficacy of these ACE2 mutants using the SARS-CoV-2 pseudoviruses. We initially used the WT pseudovirus. ACE2/WT showed an IC50 of 15.44 μg/ml (Fig. 5 and Supplementary Table 2). For ACE2 mutants, as expected, all the three mutants showed much improved entry inhibition. The determined IC50 were 1.21, 1.52, and 2.04 μg/ml for ACE2[W19/Y330], ACE2[W27/Y330], and ACE2[W19/W27/Y330], respectively (Fig. 5 and Supplementary Table 2). We also prepared the divalent protein forms for these ACE2 mutants via fusion with the Fc tag. Several recent studies have shown that ACE2 multivalency can increase both the protein stability and its entry-inhibition capacity toward SARS-CoV-2.^24,29^ Expectedly, fusion with Fc indeed further improved the inhibition efficacy. The three Fc-tagged ACE2 mutants showed an IC50 of 0.09 μg/ml for ACE2[W19/Y330]-Fc, 0.25 μg/ml for ACE2[W27/Y330]-Fc, and 0.53 μg/ml for ACE2[W19/W27/Y330]-Fc, respectively. These values represented ~29–172-fold improvement in comparison to the ACE2/WT protein (Fig. 5 and Supplementary Table 2).

In the next step, we further investigated if the entry-inhibition capacity of our ACE2 mutants might be affected by the known antibody-resistant mutations in S-RBD. Several single mutations in S-RBD, including N439K, G446V, N450K, L452R, A475V, S477N, V483A, E484K, F490L, and Y508H have been shown to lead to resistance.^31–34^ These mutant S-RBD proteins were subsequently prepared for SPR assays (Supplementary Fig. 6a–j). For each S-RBD mutant tested, ACE2[W19/Y330], ACE2[W27/Y330], and ACE2[W19/W27/Y330] exhibited ~4–20-fold higher affinities than ACE2/WT. Using the pseudoviruses containing these antibody-resistant mutations, the three ACE2 mutants expectedly showed evidently increased entry inhibition in comparison to the ACE2/WT protein (Fig. 5). These results demonstrated that the enhanced RBD binding associated with ACE2 W19, W27, and Y330 substitutions would not be affected by those S-RBD mutations attenuating antibody neutralization.

Moreover, we also investigated the decoy effect of our modified ACE2s toward SARS-CoV-2 South Africa (B.1.351) and India (B.1.617) variant strains. In S-RBD, the former carries the K417N-E484K-N501Y triple mutations and the latter bears the L452R-E484Q double mutations. Toward the S-RBDs of B.1.351 and B.1.617, ACE2[W19/Y330], ACE2[W27/Y330], and ACE2[W19/W27/Y330] all exhibited higher affinities than ACE2/WT (Supplementary Fig. 6k, l). Using the pseudoviruses of the B.1.351 and B.1.617 variants, the three ACE2 mutants expectedly showed increased entry-inhibition activities in comparison to the ACE2/WT protein (Fig. 5 and Supplementary Table 2). These results indicated that, toward these circulating SARS-CoV-2 variants, our ACE2 mutants also act as a better decoy than ACE2 WT.

### Aromatic-residue substitutions at ACE2 positions 19, 27, and 330 also increase SARS-CoV S-RBD binding

**Fig. 6:**
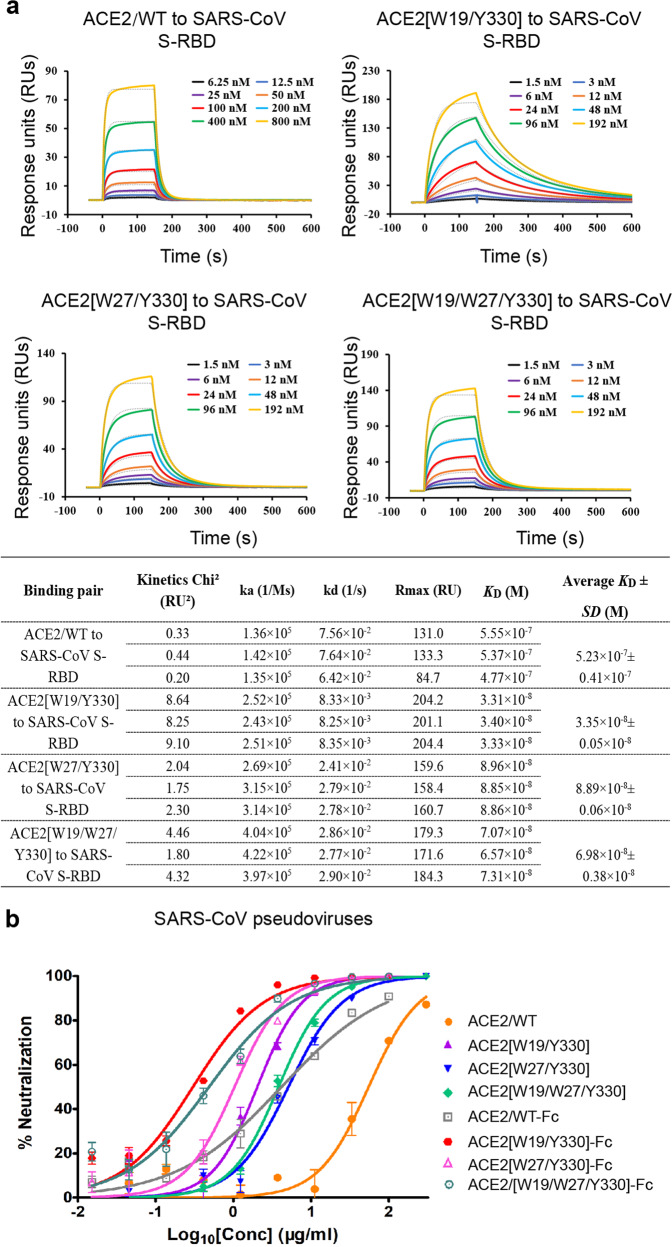
Enhanced SARS-CoV S-RBD binding and increased virus entry-inhibition associated with the ACE2 mutations. **a** An SPR assay characterizing the real-time binding kinetics of the ACE2 proteins (wild-type and mutants) to SARS-CoV S-RBD. Three independent experiments are conducted and the recorded profiles from one representative experiment are shown. The calculated kinetic parameters are summarized. **b** Increased inhibitory efficacy of the indicated ACE2 mutants in blocking virus entry of SARS-CoV. The luciferase-incorporated SARS-CoV pseudovirus is pre-incubated with the indicated ACE2 proteins (wild-type and mutants) for 1 h at 37 °C and then used to infect the human ACE2-transfecting 293T cells. Luciferase activities in target are measured. Each point represents the mean ± SD from triplicate experiments

In light of the spike-sequence similarity and the resembled S-RBD binding mode by ACE2 between SARS-CoV and SARS-CoV-2,^20,22^ we then set out to investigate if these aromatic-residue substitutions could similarly increase the ACE2 binding to SARS-CoV S-RBD. Using SPR, the binding affinity between ACE2/WT and SARS-CoV S-RBD was determined to be 523 ± 41 nM. In the parallel experiments, ACE2[W19/Y330], ACE2[W27/Y330], and ACE2[W19/W27/Y330] all showed greatly elevated binding, whose affinities were determined to be 33.5 ± 0.5 nM, 88.9 ± 0.6 nM, and 69.8 ± 3.8 nM, respectively (Fig. 6a). The efficacy of the three ACE2 mutants to inhibit SARS-CoV entry was also investigated using the SARS-CoV pseudovirus. As expected, these binding enhancement mutants exhibited elevated entry inhibition. While ACE2/WT inhibited the virus entry with an IC50 of 54.88 μg/ml, ACE2[W19/Y330], ACE2[W27/Y330], and ACE2[W19/W27/Y330] showed an IC50 of 2.10, 5.11, and 3.82 μg/ml, respectively (Fig. 6b and Supplementary Table 2). The inhibition efficacy could be further improved by fusion ACE2 with Fc, whose IC50s were determined to be 0.34 μg/ml for ACE2[W19/Y330]-Fc, 1.04 μg/ml for ACE2[W27/Y330]-Fc, and 0.49 μg/ml for ACE2[W19/W27/Y330]-Fc, respectively (Fig. 6b and Supplementary Table 2).

## Discussion

While many countries have taken great efforts to prevent the transmission of SARS-CoV-2, including practical strategies and scientific measures,^35^ it is still an urgent issue to develop effective antivirals for clinical treatment of COVID-19. Since the viral recognition of human ACE2 is a key event in SARS-CoV-2 infection, the soluble ecto-domain protein of ACE2 can in turn function as a decoy by competing against the viral engagement of ACE2 on the cell surface and consequently inhibiting virus entry.^24,28^ It is notable that ACE2 protein itself is superior in drug development. The protein has been shown to protect from severe acute lung injury in mice.^36^ In addition, recombinant ACE2 has already been tested in Phase I and II human clinical trials and its safety and good tolerance by the patients have been well documented.^37,38^ The anti-SARS-CoV-2 activity of recombinant soluble ACE2 is evident in vitro.^24–26^ Furthermore, a clinical-grade human-ACE2 protein preparation, namely APN01, has entered Phase II clinical trial to treat COVID-19 in several countries (https://www.clinicaltrials.gov/ct2/show/study/NCT04335136?term=APN01&draw=2&rank=1). The in vivo efficacy of the protein is also recently reported in a COVID-19 patient hospitalized in the Intensive Care Unit.^27^ We therefore believe that recombinant soluble ACE2 represents a promising drug candidate for COVID-19 treatment. In such a context, increasing the protein’s S-RBD binding affinity and thereby its viral entry-inhibition efficacy via limited mutations would add weight to drug development against SARS-CoV-2 based on ACE2.

In addition to ACE2 mutagenesis for sequence optimization to enhance the spike binding as illustrated in recent reports^26,28^ and in the current study, modification of ACE2 into the multivalent protein form via protein engineering represents an alternative way toward improved entry-inhibition capacity.^24,29^ It has been shown that ACE2 fused with Fc or soluble ACE2 protein containing domain CLD that is suggested to facilitate ACE2 dimerization can significantly enhance the S-RBD binding by ACE2.^24,39^ In the current study, our functional data clearly show that the binding enhancement ACE2 mutants (ACE2[W19/Y330], ACE2[W27/Y330], and ACE2[W19/W27/Y330]) can be combined with Fc-fusion to further increase its efficacy against SARS-CoV-2 entry. It is notable that a recent study has reported a feasible way of ACE2 trimerization that outperforms Fc-fusion, increasing the binding affinity between ACE2 and SARS-CoV-2 S-RBD by about 1000 fold.^29^ We therefore believe that combination of the ACE2 mutations with protein trimerization would likely lead to much better SARS-CoV-2 entry inhibitors.

Similar to other pandemic RNA viruses such as ebolavirus,^40^ SARS-CoV-2 also exhibit a high mutation rate.^41–44^ While several highly effective neutralizing antibodies have been identified and are currently tested in humans,^10,12,45–47^ antibody administration could result in the rapid occurrence of resistant mutations in viral spike.^31,32^ As a matter of fact, a series of such antibody-resistant mutations have already been identified, which are shown to severely compromise the antibody efficacy.^31–33,48^ In contrast, both the SPR data and the results of our virus entry-inhibition assay show that, toward these antibody-resistant mutants, the binding enhancement ACE2s outperform ACE2/WT in both S-RBD binding and the virus entry inhibition. In light of the fact that these antibody-resistant viruses should also engage ACE2 for virus entry, we believe the identified ACE2 mutants could act as neutralizing biologics with broader breath toward both WT and the immune-escaped viruses. Of note, the natural virus variant of SARS-CoV-2 bearing the E484K mutation in S-RBD, which is initially identified in South Africa, has drawn more and more attention.^49,50^ The variant has been shown to exhibit strong resistance to human convalescent serum antibodies and a series of monoclonal neutralizing antibodies.^48,51,52^ This mutation, however, does not compromise the neutralization efficacy of our ACE2 mutants. As a matter of fact, using the pseudoviruses of SARS-CoV-2 B.1.351 and B.1.617 variants, we have shown that our ACE2 mutants expectedly behave as a better decoy than the WT ACE2. In face of these currently-circulating SARS-CoV-2 variants and possibly of other naturally-occurred resistant variants, our binding enhancement ACE2s might be an alternative option to antibodies in terms of inhibiting the viral entry and infection.

In human populations, the *ACE2* gene is one of the most polymorphous genes. Over 10,000 different variants of human ACE2 have been recorded in dbSNP.^53^ A recent study has identified 218 single-nucleotide variations (SNVs) of ACE2 that may affect its binding to SARS-CoV-2 S-RBD.^54^ Of these SNVs, substitutions of serine by proline at position 19 (S19P) and of threonine by alanine at position 27 (T27A) are believed to occur at a population allele frequency of 0.000252 and 0.000011, respectively.^54^ In comparison to S19 and T27, P19, and A27 are residues of even smaller side-chains. They can not afford as many vdw contacts as tryptophane in our ACE2 mutants. We therefore believe that, in individuals with the S19P and T27A SNV-mutations, our modified ACE2s would also act as a better decoy.

Of the known human infective coronaviruses, SARS-CoV-2, SARS-CoV, and human coronavirus NL63 (HCoV-NL63) all engage ACE2 for cell entry.^2,13,16–18^ SARS-CoV-2 and SARS-CoV recognize the receptor in a quite similar mode. Accordingly, S19, T27, and N300 of ACE2 all locate along the S-RBD binding interface (Supplementary Fig. 10). In consistent with the structural observation, our modified ACE2 proteins exhibit increased binding capacity for both SARS-CoV-2 and SARS-CoV S-RBDs. In contrast, HCoV-NL63 engages ACE2 in a different binding mode such that ACE2 residues S19, T27, and N300 barely interact with HCoV-NL63 S-RBD (Supplementary Fig. 10). In such a context, we believe that our modified ACE2s would not benefit the spike binding for HCoV-NL63.

Of the three aromatic-residue substitutions in ACE2, W19, and W27 are separated in sequence by seven amino acids and in space by about two helix-turns. Nevertheless, our structures reveal that W19 and W27 orient their side-chains toward each other, which would lead to strong steric conflicts. It is noteworthy that W27 is accommodated in a hydrophobic pocket in SARS-CoV-2 S-RBD. In light of the large size of the tryptophan indole ring, the side-chain conformation of W27 currently observed in our structure likely represents the best match to the pocket. Such conformation, however, is not compatible with that of W19. These observations indicate that substitution T27 with hydrophobic residues with relatively smaller side-chains (e.g., leucine and isoleucine) would likely better pair with W19, which might be tested in the future.

It is also unexpected that ACE2/active-site-mutant (ACE2 with only the H374A, H378A, and E402A mutations) has been shown to exhibit slightly increased affinity toward SARS-CoV-2 S-RBD (Supplementary Fig. 3). These three active-site residues are located in the substrate-binding groove formed between subdomains I and II, which is sterically far away from the S-RBD binding interface located in subdomain I (Supplementary Fig. 7). The three residue mutations are therefore unlikely directly affecting S-RBD binding. Noted that ACE2[W19/Y330] and ACE2[W27/Y330] that also contain the active-site residue mutations are apparently captured in the closed conformation in our structures (Fig. 2d, e), we therefore propose a possibility that ACE2/active-site-mutant might have also been locked in the closed conformation and that the open/close conformation of the receptor might affect its binding to S-RBD. The well-known ACE2 inhibitor of MLN-4760 was subsequently incubated with ACE2/WT to trap the protein in the closed conformation. According to previous structural studies,^14^ MLN-4760 would occupy the substrate-binding groove, locking ACE2 in the closed state. Our differential scanning fluorimetry (DSF) data, with a melting-temperature shift of ~1.5 ℃ after the inhibitor bound (Supplementary Fig. 8a–c), further demonstrate that MLN-4760 could form stable complex with ACE2 in solution to restrict the receptor in the closed conformation. ACE2/WT in the presence or absence of MLN-4760 was subsequently analyzed for their interactions with SARS-CoV-2 and SARS-CoV S-RBDs via SPR. The affinities for ACE2 + MLN-4760 were determined to be 71.4 ± 6.2 nM toward SARS-CoV-2 S-RBD and 971 ± 108 nM toward SARS-CoV S-RBD, which represented an ~1.2-fold higher binding for the SARS-CoV-2 ligand (83.4 ± 1.9 nM) but an about 2-fold lower binding for the SARS-CoV ligand (500 ± 32 nM) than ACE2 itself. Noted that the change in the binding for SARS-CoV-2 (increase) and SARS-CoV (decrease) S-RBDs are opposite, the observed affinity difference between ACE2/WT alone and ACE2 + MLN-4760 is therefore unlikely an experimental error but, in our opinion, a reflection of ACE2’s open/close conformation slightly affecting S-RBD binding. In addition, such variance in binding was consistently observed in three independent comparison experiments. Using the unpaired *t* test, the *P* values were determined to be <0.05 for SARS-CoV-2 S-RBD and <0.01 for SARS-CoV S-RBD, demonstrating that the observed affinity difference was of statistical significance (Supplementary Fig. 9a, b). If the close conformation of ACE2 could increase SARS-CoV-2 S-RBD binding, though with a rather limited enhancement, an accumulative effect of such enhancement in the binding and thereby in the virus entry during a long time of disease course as in the infected patients might be non-negligible. As a matter of fact, hypertension has been identified as one of the major risk factors associating with the severity and fatality of SARS-CoV-2 infection.^27,55,56^ The hypertension patients normally would have higher levels of angiotensin II, which is the natural substrate for ACE2.^57,58^ During catalysis, angiotensin II could bind to ACE2, transiently trapping the receptor in the closed conformation. This seems to be able to build up a logical connection between hypertension and the high risk of progressing into severe diseases in SARS-CoV-2 infected patients. In support of this, the meta-analyses have revealed that the use of ACE inhibitors (that would decrease the angiotensin II level of the hypertension patients) might not increase the susceptibility of SARS-CoV-2 infection but the use of angiotensin receptor blockers (which would not decrease the angiotensin II level in hypertension patients) could augment the SARS-CoV-2 infection.^59^

## Materials and methods

### Gene cloning, protein expression, and purification

The His-tagged-proteins (ACE2 wild-type and mutants, SARS-CoV-2 and SARS-CoV S-RBDs) were prepared from insect cells using the Bac-to-Bac baculovirus expression system (Invitrogen). The original genes for SARS-CoV-2 spike (GenBank accession number MN908947.3), SARS-CoV spike (GenBank accession number AY278554.2), and human ACE2 (GenBank accession number BAB40370.1) were all synthesized in pFastBac1 vector via the EcoR I and Hind III restriction sites at Convenience Biology. For ACE2 preparation, the coding sequence for human ACE2 peptidase domain (PD) (residues 19–615) was subcloned into the pFastBac1 vector via the EcoR I and Hind III restriction sites. An N-terminal GP67 signal peptide and a C-terminal 6×His tag were added to facilitate protein secretion and purification, respectively.^60,61^ ACE2 mutants containing S19W, T27W, H34E, G326W, G326K, N330Y, K353R, H374A/H378A/E402A, S19W/T27W/H374A/H378A/E402A, S19W/N330Y/H374A/H378A/E402A, T27W/N330Y/H374A/H378A/E402A, and S19W/T27W/N330Y/H374A/H378A/E402A were subsequently generated via site-directed mutagenesis. For S-RBD preparation (SARS-CoV-2 spike residues 320-537 and SARS-CoV spike residues 307-523), S-RBD was designed to fuse in a tandem manner with an N-terminal GP67 signal peptide to guarantee protein secretion, a Trx tag to facilitate protein folding, a 6xHis tag to facilitate protein purification, and finally, an Enterokinase (EK) cleavage cite for tag removal. The above-designed coding sequences were amplified and subcloned into the pFastBac1 vector via the BamH I and Hind III restriction sites. SARS-CoV-2 S-RBD mutants containing N439K, G446V, N450K, L452R, A475V, S477N, V483A, E484K, F490L, Y508H, double mutations L452R-E484Q (as in the B.1.617 variant), and triple mutations K417N-E484K-N501Y (as in the B.1.351 variant) were then generated via site-directed mutagenesis. Each sequencing-verified plasmid was subsequently transformed into *E*. *coli* DH10Bac cells to generate the recombinant bacmids, which were then transfected into sf9 cells for further virus amplification and protein production.

For protein purification, both ACE2 (wild-type and mutants) and S-RBD proteins (wild-type and mutants) were initially purified by affinity chromatography with a 5-ml His-Trap excel column (GE Healthcare). The S-RBD proteins eluted from the His-Trap column were further cleaved using EK protease at a ratio of 1U:0.5 mg to remove the Trx and His tags. EK protease is a gift from the laboratory of Li Yang, Sichuan University. Subsequently, each target protein was loaded on a Source 15Q column (GE Healthcare) for ion exchange and then a Superdex 200 Increase 10/300 GL column (GE Healthcare) for gel filtration. The purify of the final protein preparation was determined by polyacrylamide gel electrophoresis.

We also prepared the Fc-tagged ACE2 proteins using the 293T cells. Human ACE2 (GenBank accession number BAB40370.1) was synthesized in pCAGGS vector via the KpnI and XhoI restriction sites at Convenience Biology. The ACE2 peptidase domain coding sequence (wild-type and mutants) was firstly in-frame combined at the N-terminus with the coding sequence for IL2 signal peptide (GenBank accession number AAH66256.1, amino acids 1–20) and at the C-terminus with the coding fragment for human Fc (GenBank accession number AAA02914.1, amino acids 250–476), and then subcloned into the pCAGGS vector via the KpnI and XhoI restriction sites. The recombinant plasmids were subsequently verified via sequencing and transiently transformed into 293T cells for protein expression and secretion. The fusion proteins were then purified via affinity chromatography using protein A-Sepharose (GE Healthcare) from the harvested cell-culture supernatants.

To prepare the S-RBD/ACE2-mutant complex, ACE2[W19/Y330] and ACE2[W27/Y330] were individually incubated with wild-type SARS-CoV-2 S-RBD at a molar ratio of 1:1.2 overnight on ice. The mixture was then subjected to gel filtration using a Superdex 200 Increase 10/300 GL column (GE Healthcare). Fractions containing the complex were pooled, characterized by polyacrylamide gel electrophoresis, and concentrated to 10 mg/ml for crystallization.

### Crystallization

All the crystals were obtained with the vapor-diffusion sitting-drop method by mixing 1 μl protein with 1 μl reservoir solution and then equilibrating against 90 μl reservoir solution at 18 °C. The initial crystallization screenings were carried out using the commercial crystallization kits (Hampton Research and Molecular Dimensions). The conditions that yield crystals were then optimized. Diffractable crystals of the SARS-CoV-2 S-RBD/ACE2[W19/Y330] complex were obtained in a condition consisting of 1.6 M ammonium sulfate, 0.1 M MES [pH 6.5], and 10% v/v 1,4-Dioxane with a protein concentration of 10 mg/ml. For the SARS-CoV-2 S-RBD/ACE2[W27/Y330] complex, the crystals were grown in a condition consisting of 2.0 M ammonium sulfate, 0.1 M sodium HEPES [pH 7.5], and 2% v/v PEG400 with a protein concentration of 10 mg/ml.

### Data collection and structure determination

For data collection, all crystals were flash-cooled in liquid nitrogen after a brief soaking in reservoir solution with the addition of 20% (v/v) glycerol. Diffraction data were collected at Shanghai Synchrotron Radiation Facility (SSRF) beamline BL18U.^62^ All data were processed with HKL2000^63^ for indexing, integration, and scaling. Structures were determined by the molecular replacement method using the Phaser program^64^ in the CCP4 suite.^65^ The structure of SARS-CoV-2 S-RBD/ACE2 complex (PDB code: 6LZG) was used to serve as the search model. Initial restrained rigid-body refinement was performed using REFMAC5,^66^ which was followed by manual rebuilding and adjustment in COOT.^63^ Further refinement was carried out using Phenix.^67^ The stereochemical qualities of the final models were assessed through the program PROCHECK.^68^ The final data processing and structure refinement statistics are summarized in Supplementary Table 1. All structural figures were generated using PyMOL (http://www.pymol.org).

### Surface plasmon resonance (SPR) experiments

All the SPR experiments were performed with the Biacore 8K system (GE Healthcare). SARS-CoV-2 S-RBD (wild-type and mutants) and SARS-CoV S-RBD (wild-type) were individually immobilized to the CM5 sensorchip (GE Healthcare) to a level of ~600 response units (RUs). Serial dilutions of the ACE2 proteins (wild-type and mutants) were flowed at 30 μl/min over S-RBD for kinetic determination. The running buffer contains 10 mM HEPES pH 7.4, 150 mM NaCl, and 0.05% Tween 20. The resulting data were fit to a 1:1 binding model using the Biacore Evaluation Software (GE Healthcare). For each binding pair, three independent kinetic assays were conducted, and the calculated kinetic parameters are summarized.

To learn if the open/close conformation of ACE2 might affect S-RBD binding, we further incubated wild-type ACE2 with MLN-4760 at a molar ratio of 1:5 for 1 h on ice to prepare the ACE2 + MLN-4760 complex. Gradient concentrations of ACE2 and ACE2 + MLN-4760 complex were then flowed over SARS-CoV-2 and SARS-CoV S-RBDs on the chip-surface at 30 μl/min. Serial dilutions of the MLN-4760 inhibitor alone were also flowed over S-RBD to exclude the possibility that unwanted interactions between MLN-4760 and S-RBD might exist. The calculated kinetic parameters are summarized and the statistical analysis was performed by GraphPad Prism 5.0 using the unpaired *t* test with data obtained from three independent experimental replicates.

### Neutralization assay

HEK-293T cells were maintained in DMEM with 10% fetal bovine serum and 1% penicillin/streptomycin. The original genes for SARS-CoV-2 spike (GenBank accession number MN908947.3) and SARS-CoV spike (GenBank accession number AY278554.2) were synthesized at Convenience Biology to include a C-terminal flag-tag coding sequence and then individually subcloned into the pCAGGS vector via the EcoR I and Bgl II restriction sites. SARS-CoV-2 spike mutants containing the antibody-resistant mutations, including N439K, G446V, N450K, L452R, A475V, S477N, V483A, E484K, F490L, and Y508H, were then generated via site-directed mutagenesis. The subsequent spike-expression plasmids were then co-transfected with a plasmid encoding an Env-defective, luciferase-expressing HIV-1 genome (pNL4-3.luc.RE) into 293T cells.^69^ Supernatants containing SARS-CoV-2 (wild-type and mutants) and SARS-CoV pseudoviruses were harvested 48 h post transfection. The pseudoviruses of B.1.351 and B.1.617 were purchased from Genomeditech company (#GM-0220PV32 and # GM-0220PV43, respectively).

Pseudotyped reporter virus assays were conducted with the HEK-293T cells that stably express human ACE2 (HEK293T-ACE2). The HEK293T-ACE2 stable cell line was prepared as previously described.^70^ In brief, HEK293T cells were transfected with a lentivirus vector pCDH encoding human ACE2 and puromycin selection marker. Puromycin-resistant cells were subsequently expanded and verified by FACS for ACE2 expression. For pseudovirus infection, HEK293T-ACE2 cells were plated into 96-well cell-culture plates with 1 × 10^4^ cells/well and cultured at 37 °C with 5 % CO_2_. The supernatants containing pseudovirus were pre-incubated with serially diluted ACE2 proteins at 37 °C for 60 min before addition to the HEK293T-ACE2 cells. The culture was refed with fresh DMEM medium 24 h after infection and incubated for an additional 48 h. Luciferase activity was measured using the One-Lumi^TM^ II Firefly Luciferase Assay Kit according to the manufacturer’s instructions (Beyotime). The relative luminescence signals (RLU) from the negative control wells (pseudovirus alone) were normalized and used to calculate neutralization percentage, which was subsequently plotted against ACE2 concentration for IC50 determination.

### Differential scanning fluorimetry (DSF) assay

The DSF assay was performed as reported.^71^ SYPRO Orange dye (Sigma) was used to probe protein thermal denaturation. Wild-type ACE2 was incubated with MLN-4760 at a molar ratio of 1:5 for 1 h on ice and loaded on a Superdex 200 Increase 10/300 GL column (GE Healthcare) for buffer exchange to remove excessive MLN-4760 inhibitor. For the DSF assay, 10 µl of the sample (wild-type ACE2 alone and ACE2 + MLN-4760 in a buffer consisting of 20 mM Tris pH 8.0, 150 mM NaCl, at a protein concentration of 20 μM) was heated, by using a linear temperature-gradient of 25–95 °C in 65 min using CFX Connect Real-Time System (Bio-Rad). Fluorescence signal as a function of temperature was monitored continuously. Each sample was measured in triplet and fitted with the Boltzmann equation using GraphPad Prism 5.0 (GraphPad Software).

## Supplementary information

Supplementary materials

## Data Availability

The structural factors and the atomic coordinates for the SARS-CoV-2 S-RBD/ACE2[W19/Y330] and SARS-CoV-2 S-RBD/ACE2[W27/Y330] complex structures have been deposited into the Protein Data Bank with the accession number of 7EFP and 7EFR, respectively.
