# Supplementary material for: S19W, T27W, and N330Y mutations in ACE2 enhance SARS-CoV-2 S-RBD binding toward both wild-type and antibody-resistant viruses and its molecular basis
Source: Signal Transduct Target Ther. 2021 Sep 16;6:343. doi: 10.1038/s41392-021-00756-4 (PMC8444507; doi:10.1038/s41392-021-00756-4)
Supplement: Supplementary file 1 — Supplementary materials [file 41392_2021_756_MOESM1_ESM.docx]

Supplementary Materials for

S19W, T27W, and N330Y mutations in ACE2 enhance SARS-CoV-2 S-RBD binding toward both wild-type and antibody-resistant viruses and its molecular basis

Fei Ye^1,†^, Xi Lin^1,†^, Zimin Chen^1^, Fanli Yang^1^, Sheng Lin^1^, Jing Yang^1^, Hua Chen^1^, Honglu Sun^1^, Lingling Wang^1^, Ao Wen^1^, Xindan Zhang^1^, Yushan Dai^1^, Yu Cao^1,2^, Jingyun Yang^3^, Guobo Shen^3^, Li Yang^3,^, Jiong Li^3^, Zhenling Wang^3,^, Wei Wang^3^, Xiawei Wei^3^, Guangwen Lu^1,*^

Correspondence to: lugw@scu.edu.cn

**This PDF file includes:**

Figures. S1 to S10

Tables S1 to S3


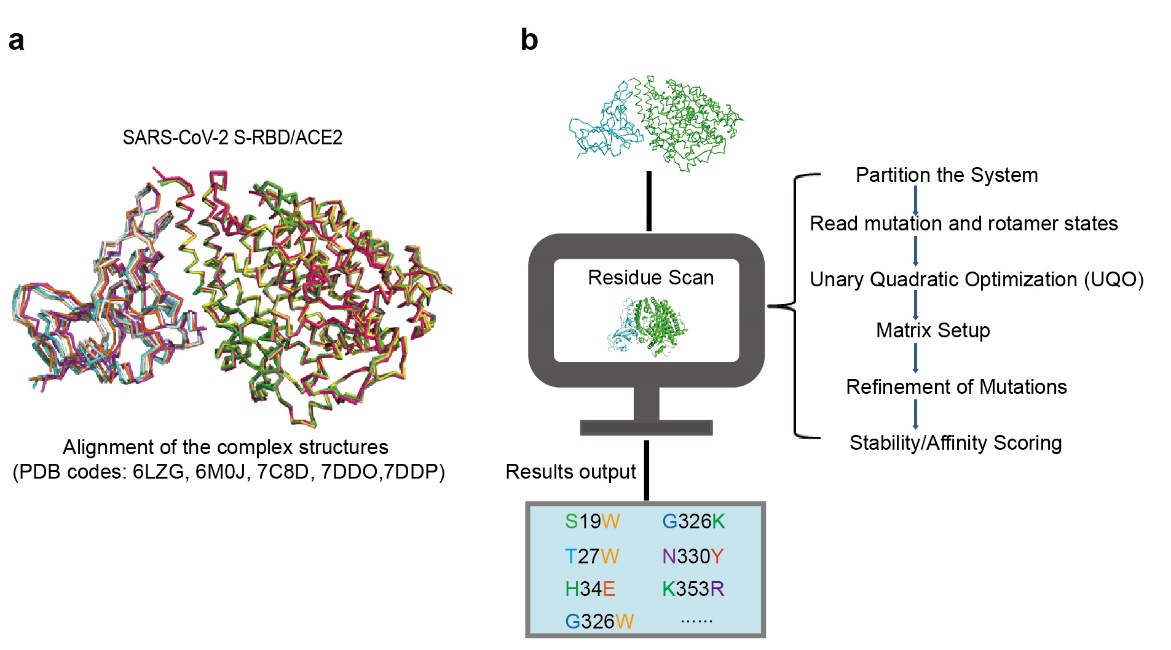
**Figure. S1**

**Figure. S1. A computer-aided MOE-based virtual screening in ACE2 toward enhanced S-RBD binding based on previously reported SARS-CoV-2 S-RBD/ACE2 complex structures.** (a) Highly conserved binding interface between SARS-CoV-2 S-RBD and ACE2 revealed by superimposition of reported complex structures (PDB codes: 6LZG, 6M0J, 7C8D, 7DDO and 7DDP). The structures are shown as ribbons and colored as indicated (6LZG: ACE2 in green and S-RBD in cyan, 6M0J: ACE2 in yellow and S-RBD in wheat, 7C8D: ACE2 in hot-pink and S-RBD violet, 7DDO: ACE2 in light-orange and S-RBD in orange, 7DDP: ACE2 in grays and S-RBD in white). (b) The schematic of MOE-based virtual screening process. The result of mutation options is listed.

**Figure. S2**


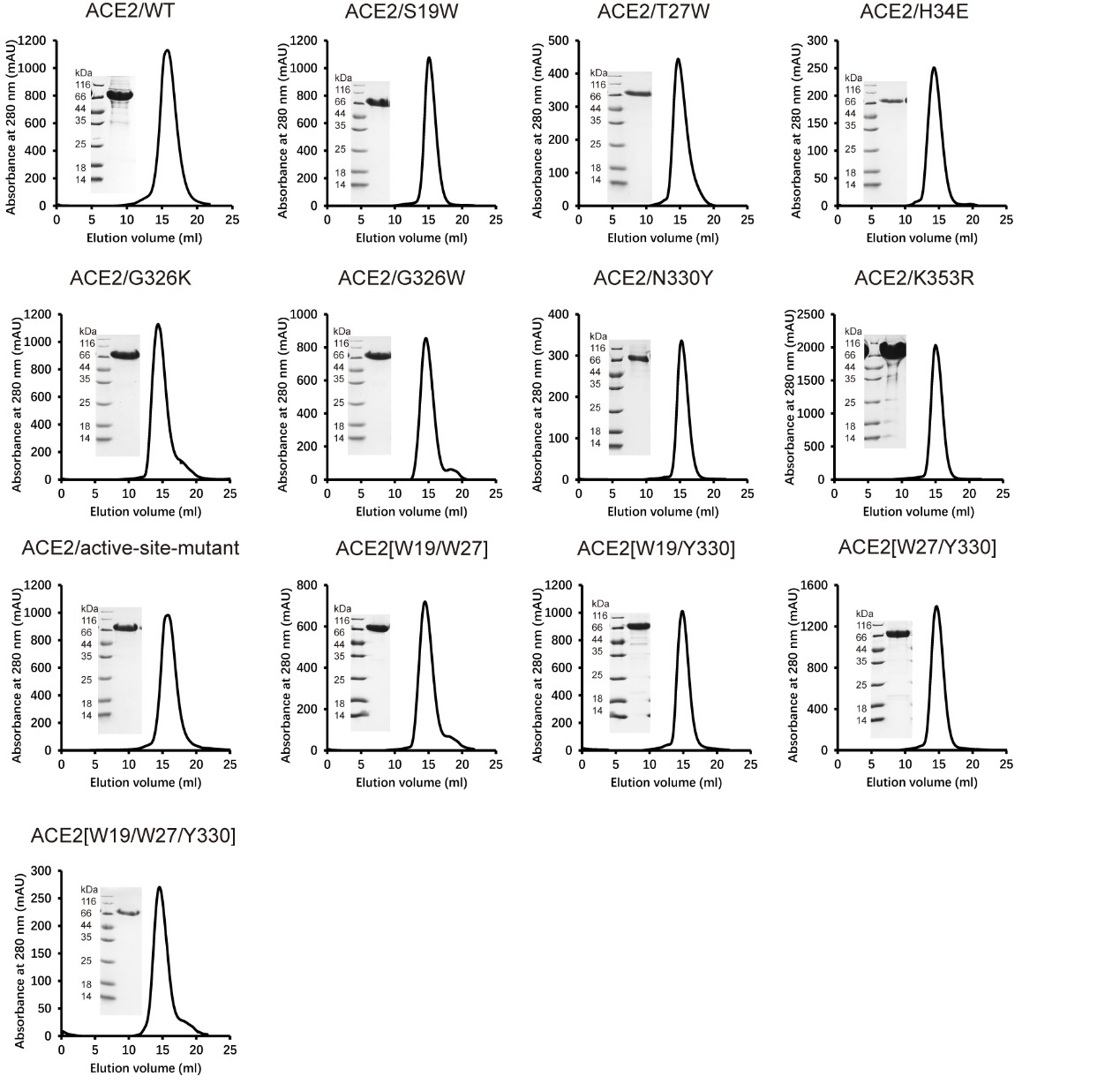


**Figure. S2. Solution behaviors of the indicated ACE2 proteins (wild-type and mutants) on a Superdex 200 Increase 10/300 GL column.** The 280-nm absorbance chromatograph and the SDS-PAGE migration profile of the proteins are shown. ACE2/WT: wild-type ACE2; ACE2/S19W: ACE2 containing the S19W mutation; ACE2/T27W: ACE2 containing the T27W mutation; ACE2/H34E: ACE2 containing the H34E mutation; ACE2/G326K: ACE2 containing the G326K mutation; ACE2/G326W: ACE2 containing the G326W mutation; ACE2/N330Y: ACE2 containing the N330Y mutation; ACE2/K353R: ACE2 containing the K353R mutation; ACE2/active-site-mutant: ACE2 containing the H374A, H378A, and E402A mutations; ACE2[W19/W27]: ACE2 containing the S19W, T27W, H374A, H378A, and E402A mutations; ACE2[W19/Y330]: ACE2 containing the S19W, N330Y, H374A, H378A, and E402A mutations; ACE2[W27/Y330]: ACE2 containing the T27W, N330Y, H374A, H378A, and E402A mutations; ACE2[W19/W27/Y330]: ACE2 containing the S19W, T27W, N330Y, H374A, H378A, and E402A mutations.


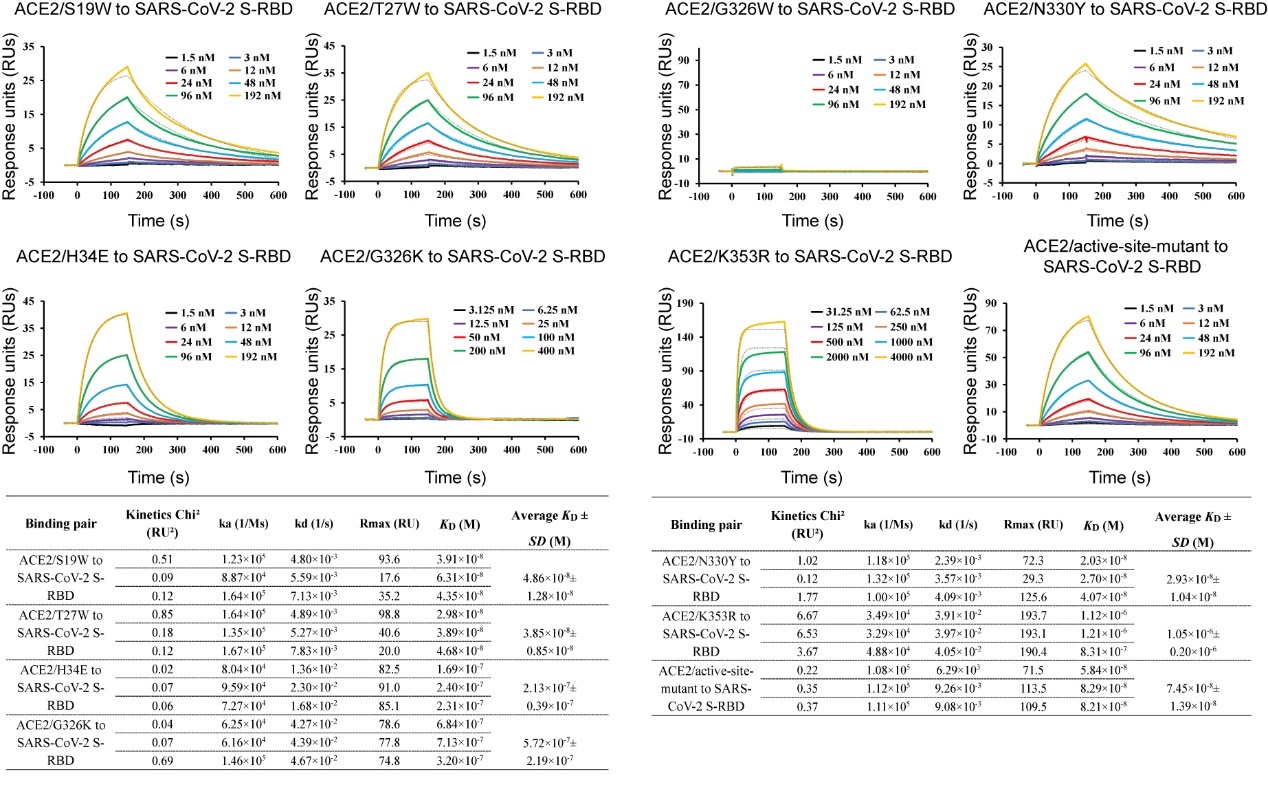
**Figure. S3**

**Figure. S3. An SPR assay characterizing the real-time binding kinetics of** **the indicated ACE2 mutant proteins to SARS-CoV-2 S-RBD.** Three independent experiments are conducted and the recorded proﬁles from one representative experiment are shown. The slow-on/slow-off kinetic data are analyzed by the 1:1 binding model. The calculated kinetic parameters are summarized. ACE2/S19W: ACE2 containing the S19W mutation; ACE2/T27W: ACE2 containing the T27W mutation; ACE2/H34E: ACE2 containing the H34E mutation; ACE2/G326K: ACE2 containing the G326K mutation; ACE2/G326W: ACE2 containing the G326W mutation; ACE2/N330Y: ACE2 containing the N330Y mutation; ACE2/K353R: ACE2 containing the K353R mutation; ACE2/active-site-mutant: ACE2 containing the H374A, H378A, and E402A mutations.


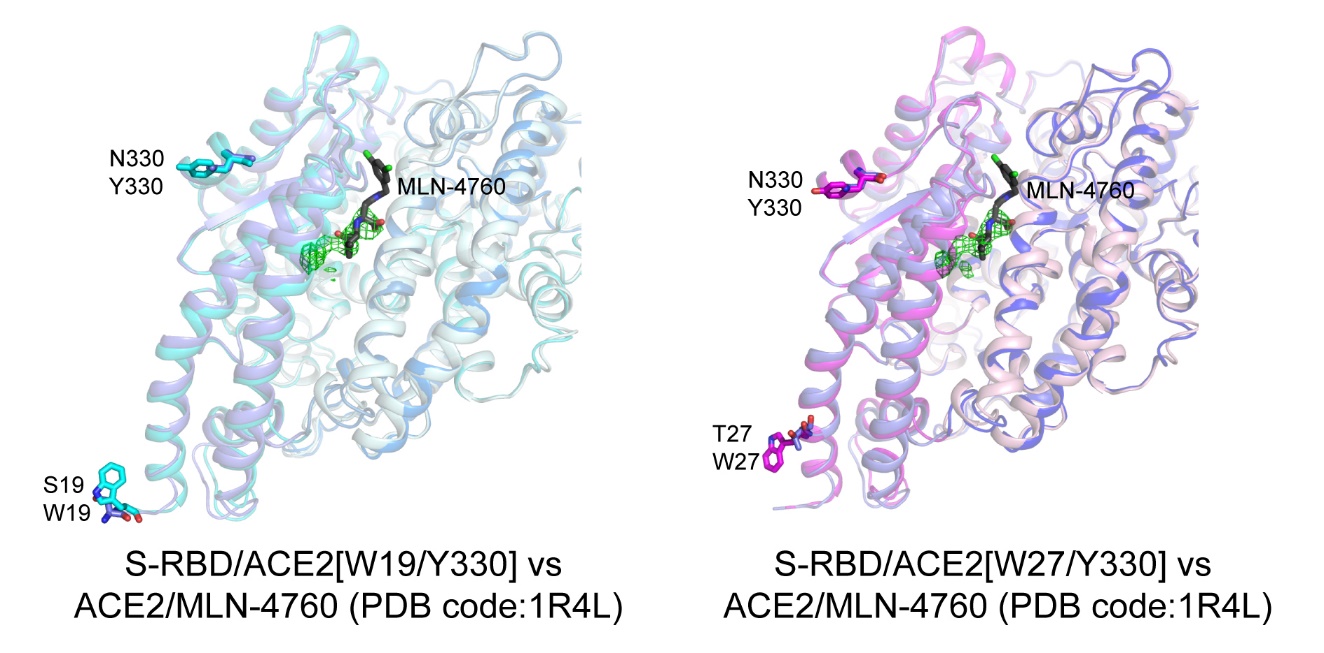
**Figure. S4**

**Figure. S4. The ACE2-active-site-located electron densities in our S-RBD/ACE2[W19/Y330] and S-RBD/ACE2[W27/Y330] complex structures are partially overlapped with the MLN-4760 inhibitor in the ACE2/MLN-4760 complex structure (PDB code: 1R4L).** The electron densities in our structures are contoured at 1.5 σ using the｜Fo｜-｜Fc｜map and colored green. The MLN-4760 inhibitor is shown as sticks. The mutated W19, W27 and Y330 residues, which are far away from the ACE2 active-site cleft, are highlighted by sticks and labeled.


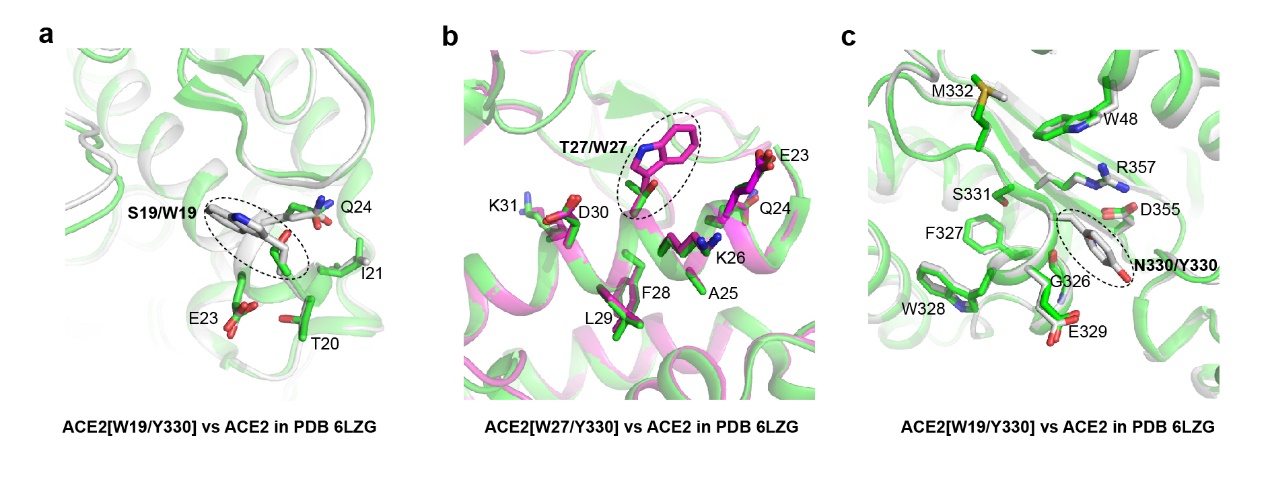
**Figure. S5**

**Figure. S5. The S19W, T27W, and N330Y mutations in ACE2 do not induce obvious conformational changes to the neighboring interface residues in the receptor.** The neighboring residues are defined as those ACE2 amino acids located within a distance of ≤4.5 Å from S19, T27, and N330, based on a previously reported SARS-CoV-2 S-RBD/ACE2 complex structure with a PDB code of 6LZG. The corresponding amino acids in our structures are then selected for superimposition. Clearly shown is that all these residues could be well-aligned. (a) Superimposition of our S-RBD/ACE2[W19/Y330] structure (in gray) onto the S-RBD/ACE2 structure (PDB code: 6LZG, in green) focusing on S19/W19 and its neighboring residues. (b) Superimposition of our S-RBD/ACE2[W27/Y330] structure (in magenta) onto the S-RBD/ACE2 structure (PDB code: 6LZG, in green) focusing on T27/W27 and its neighboring residues. (c) Superimposition of our S-RBD/ACE2[W19/Y330] structure (in gray) onto the S-RBD/ACE2 structure (PDB code: 6LZG, in green) focusing on N330/Y330 and its neighboring residues.

**Figure. S6**


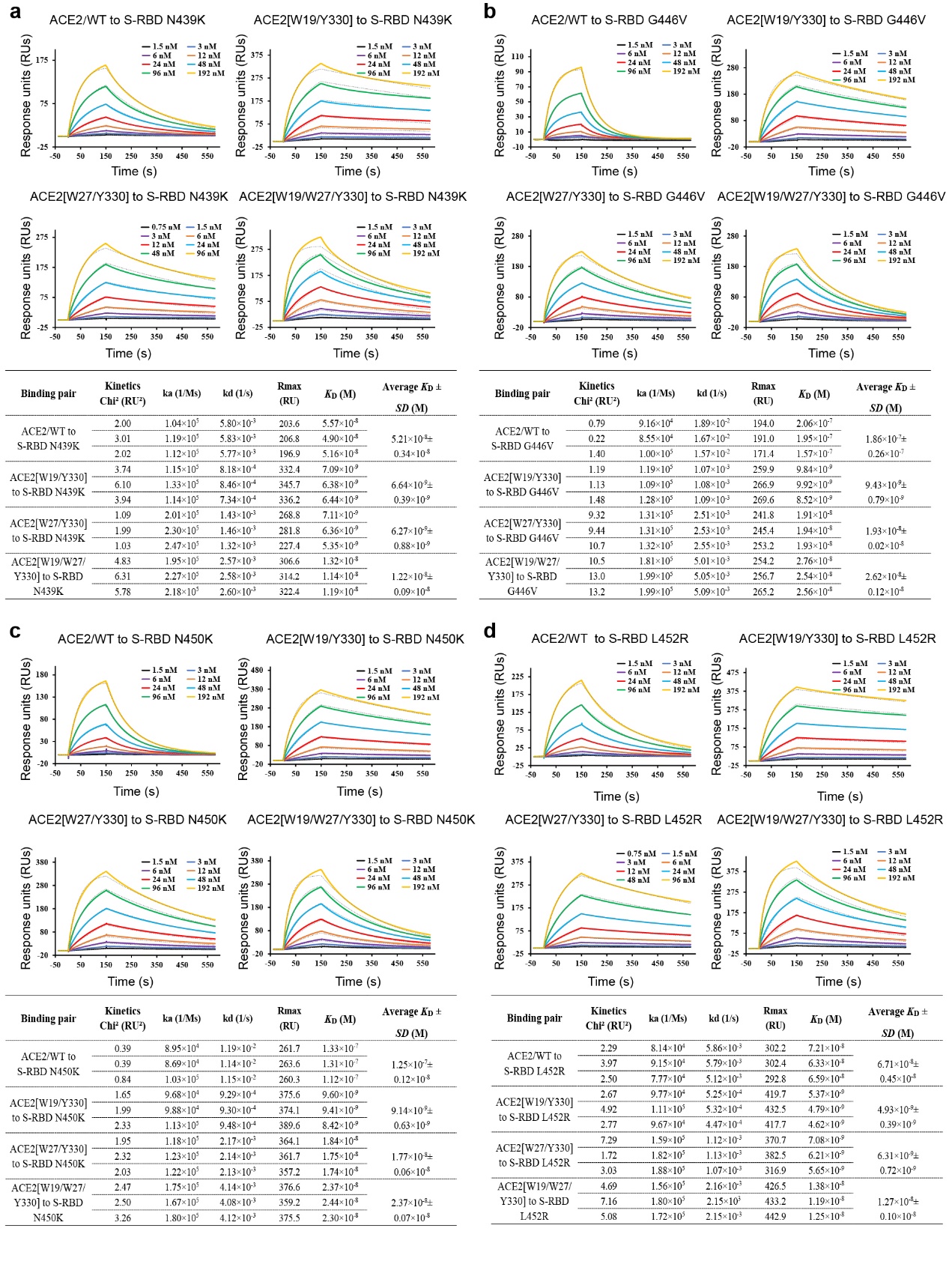


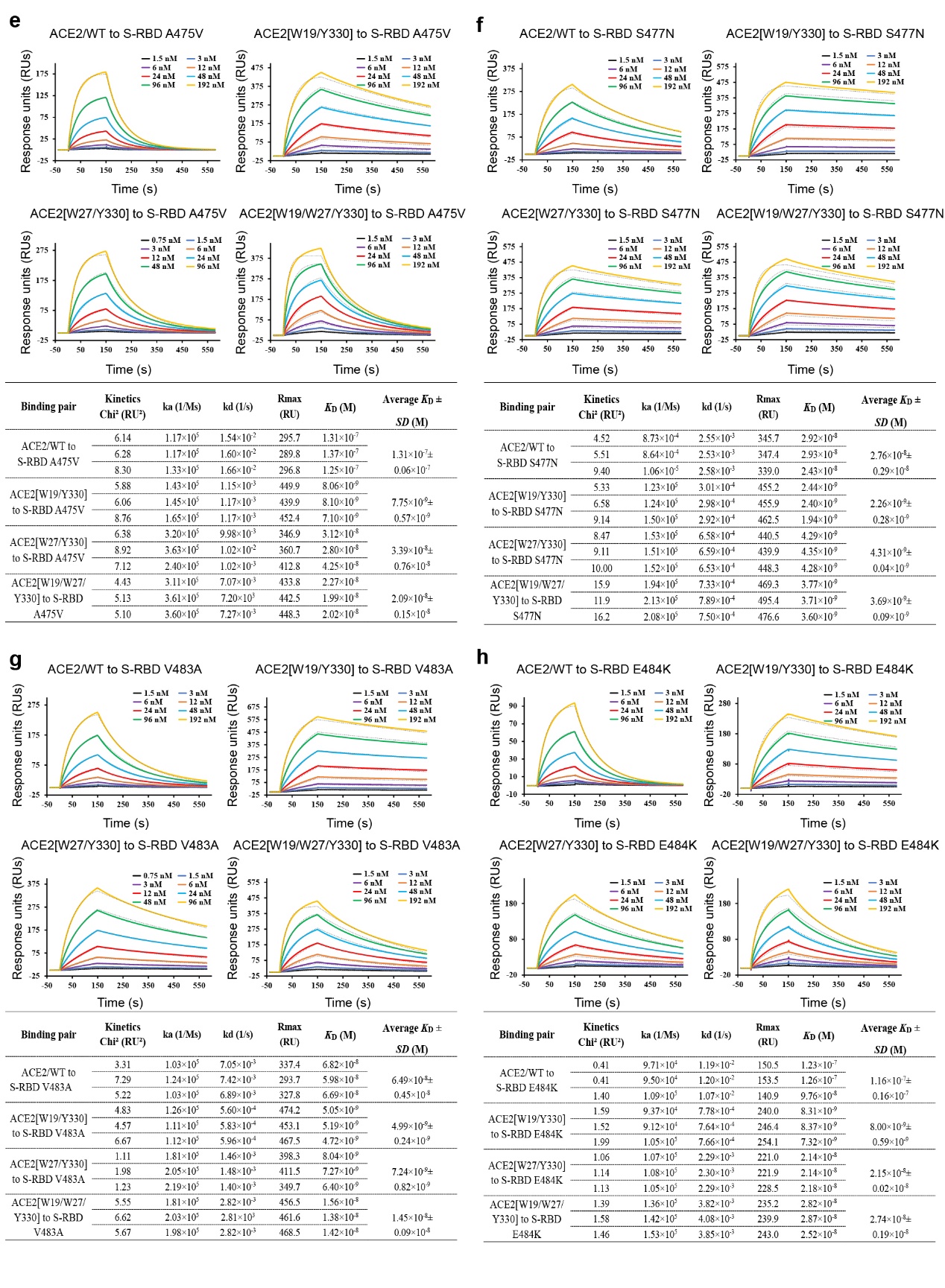


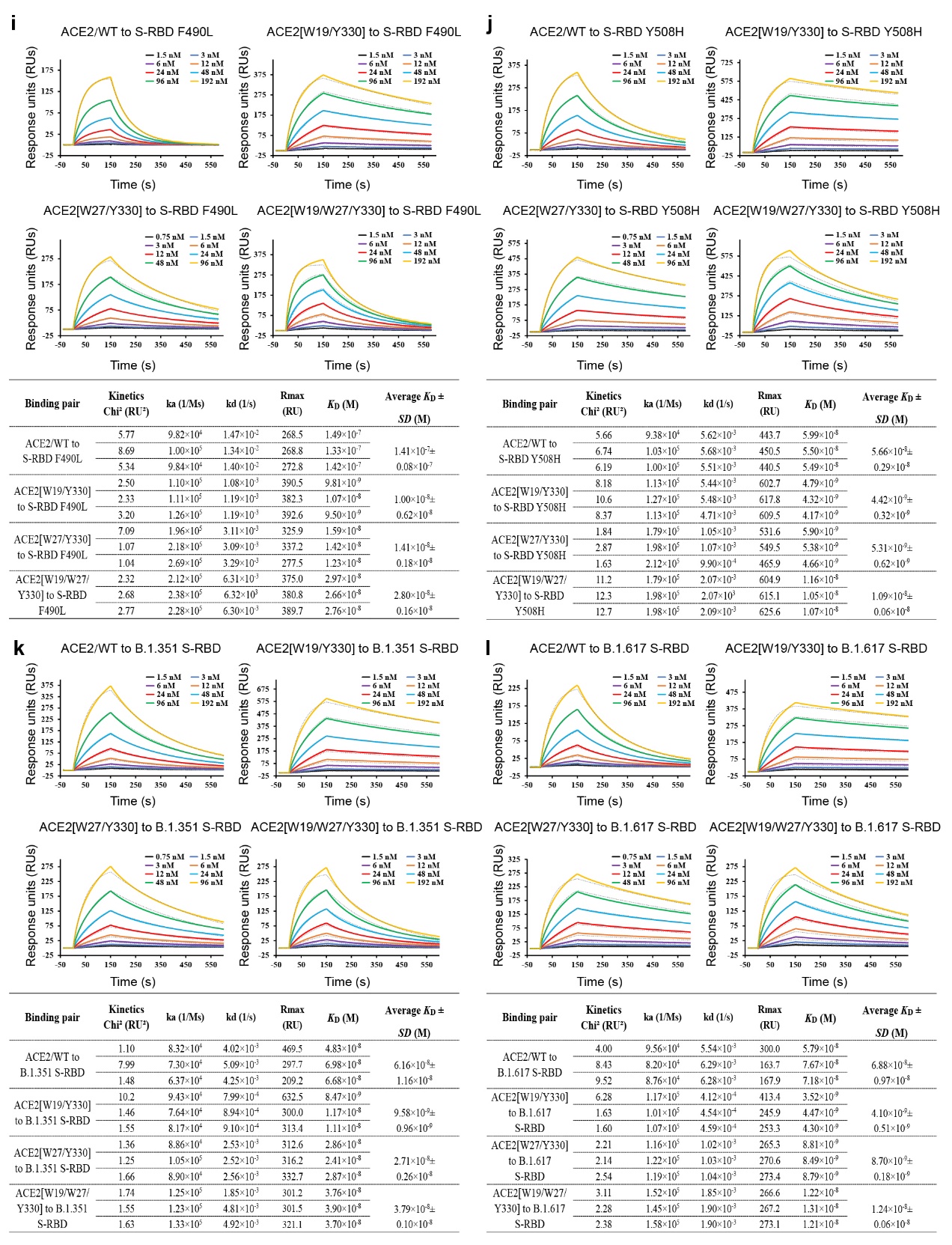


**Figure. S6. An SPR assay characterizing the real-time binding kinetics of the ACE2 proteins (wild-type and mutants) to SARS-CoV-2 S-RBD harboring the indicated antibody-resistant mutations and the mutations present in the circulating virus variants.** For each binding pair, three independent experiments are conducted and the recorded proﬁles from one representative experiment are shown. The slow-on/slow-off kinetic data are analyzed by the 1:1 binding model. The calculated kinetic parameters are summarized. (a) The binding of the indicated ACE2s to SARS-CoV-2 S-RBD containing the N439K mutation. (b) Binding of ACE2s to S-RBD G446V. (c) Binding of ACE2s to S-RBD N450K. (d) Binding of ACE2s to S-RBD L452R. (e) Binding of ACE2s to S-RBD A475V. (f) Binding of ACE2s to S-RBD S477N. (g) Binding of ACE2s to S-RBD V483A. (h) Binding of ACE2s to S-RBD E484K. (i) Binding of ACE2s to S-RBD F490L. (j) Binding of ACE2s to S-RBD Y508H. (k) Binding of ACE2s to the B.1.351 S-RBD. (l) Binding of ACE2s to the B.1.617 S-RBD.


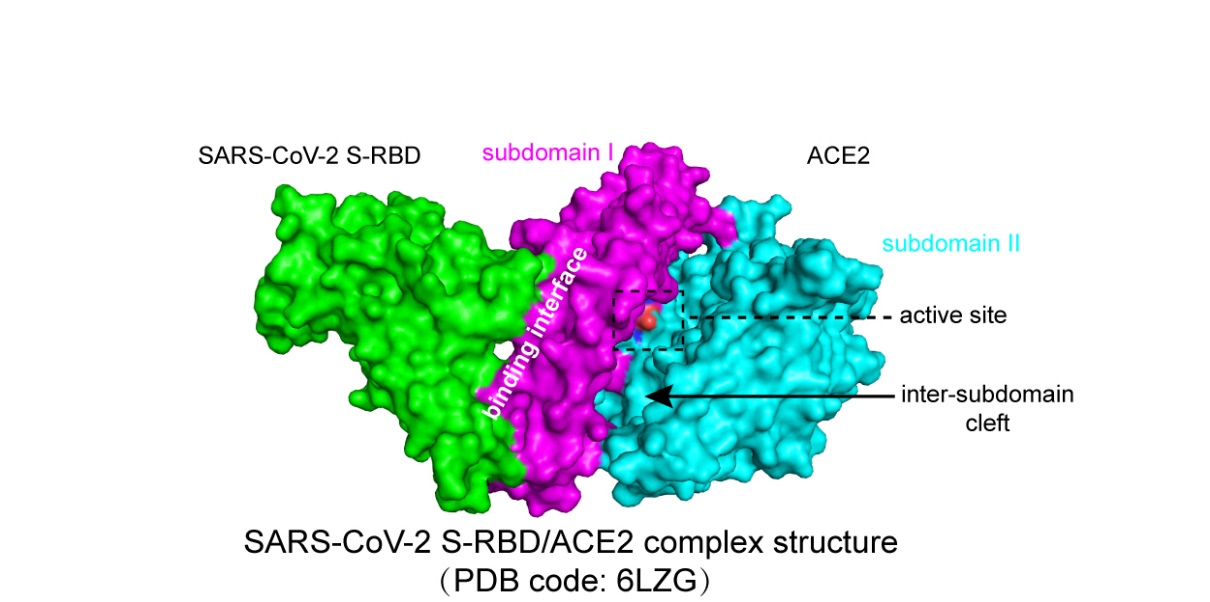
**Figure. S7**

**Figure. S7. An overview of the SARS-CoV-2 S-RBD/ACE2 complex structure (PDB code: 6LZG) shown in surface.** S-RBD is colored green and ACE2 subdomains I and II are in magenta and cyan, respectively. The S-RBD/ACE2 binding interface, the inter-subdomain cleft and the catalytic active site in ACE2 are highlighted and labeled. Clearly shown is that the location of the active-site residues of ACE2 is sterically far away from the S-RBD binding interface.

**Figure. S8**

**
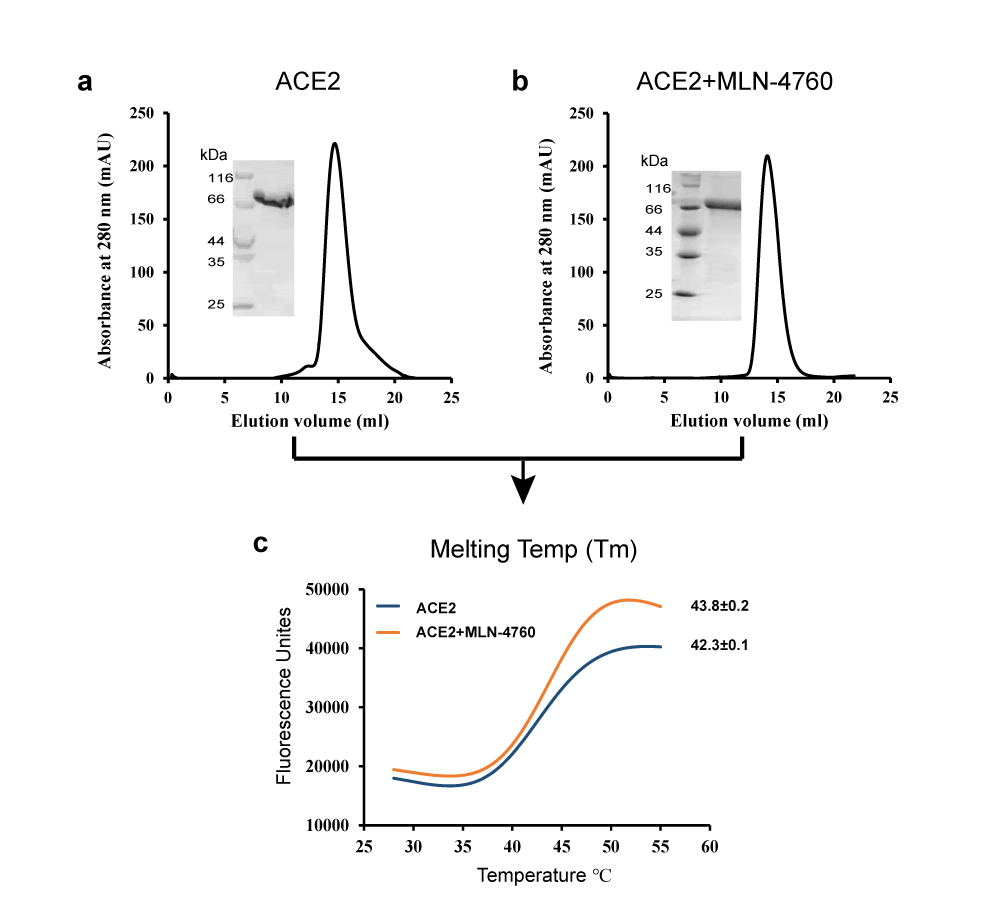
**

**Figure. S8. Binding of MLN-4760 to wild-type ACE2 to form a stable complex as characterized by differential scanning fluorimetry (DSF).** The ACE2 protein is pre-incubated with MLN-4760 at a molar ratio of 1:5, further purified to remove the excessive inhibitor, and finally applied for DSF test in parallel with the ACE2 protein alone. The recorded gel-filtration and DSF profiles are shown. (a) The separation profile of the ACE2 protein alone on a Superdex 200 Increase 10/300 GL column. (b) The separation profile of the ACE2+MLN-4760 complex on a Superdex 200 Increase 10/300 GL column. The inset figures show the SDS-PAGE migration-profiles of the pooled samples. (c) Representative DSF curves for the ACE2 protein alone and the ACE2+MLN-4760 complex.


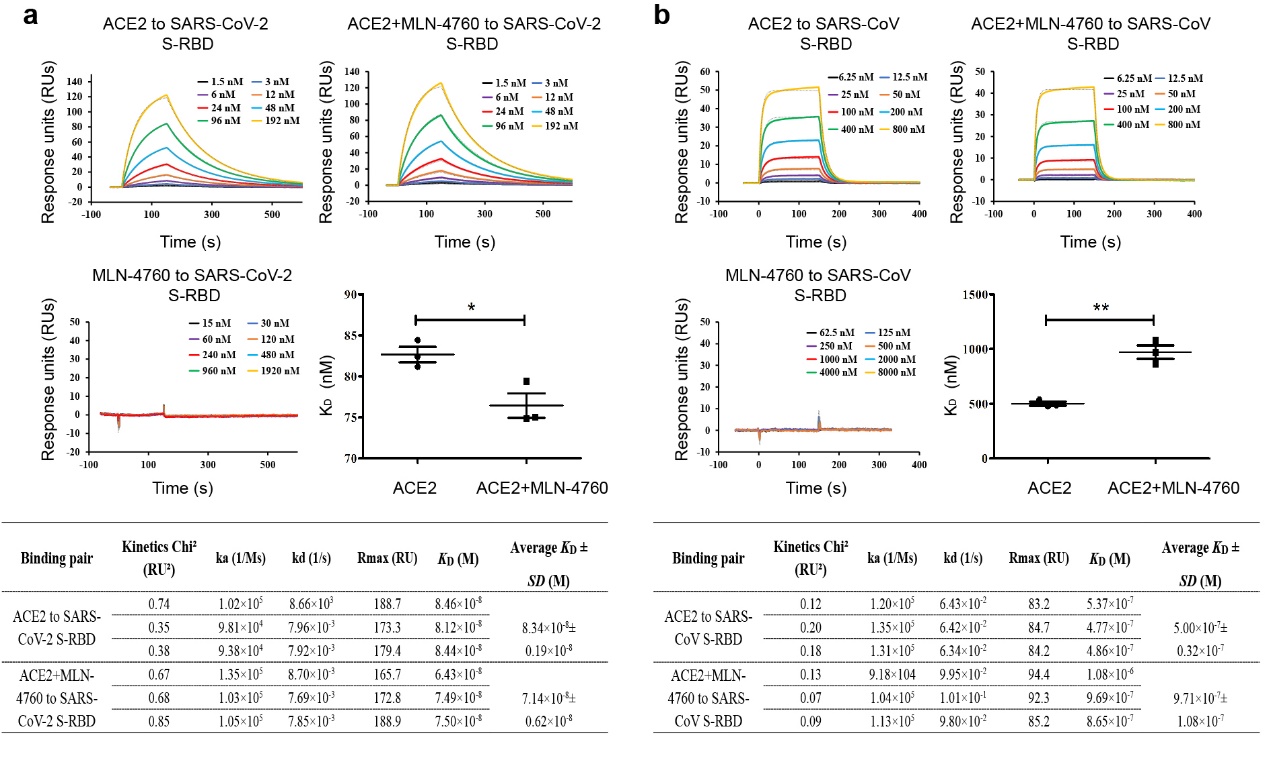
**Figure. S9**

**Figure. S9. Evidence of ACE2’s open/close conformation affecting S-RBD binding as characterized by SPR.** Gradient concentrations of ACE2 (wild-type ACE2 alone), ACE2+MLN-4760 (wild-type ACE2 in complex with MLN-4760), and MLN-4760 (the inhibitor alone) are flowed over SARS-CoV-2 and SARS-CoV S-RBDs on the chip-surface in parallel. The recorded proﬁles from one representative experiment (out of three independent experiments) are shown and the calculated kinetic parameters are summarized. The results from three independent experiments are also analyzed using the unpaired *t* test. * indicates *P*﹤0.05 and ** indicates *P*﹤0.01. (a) Binding of ACE2 in the presence or absence of MLN-4760 to SARS-CoV-2 S-RBD. (b) Binding of ACE2 in the presence or absence of MLN-4760 to SARS-CoV S-RBD.


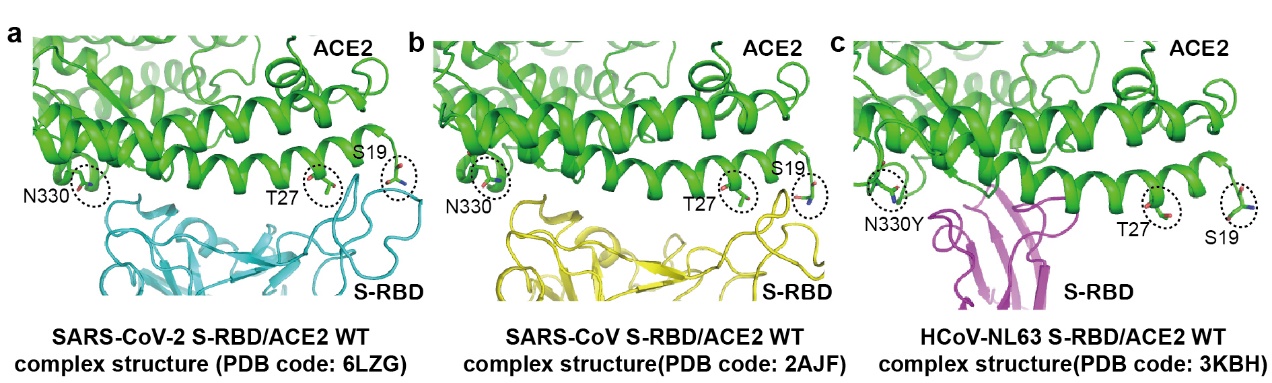
**Figure. S10**

**Figure. S10. ACE2 amino acids S19, T27 and N330 locate in the SARS-CoV-2 and SARS-CoV S-RBD binding interface but not in the HCoV-NL63 S-RBD binding interface.** S19, T27 and N330 of ACE2 are shown as sticks and highlighted by circling with dashed lines. (a) The binding interface between SARS-CoV-2 S-RBD (cyan) and ACE2 (green) based on PDB code: 6LZG. (b) The binding interface between SARS-CoV S-RBD (yellow) and ACE2 (green) based on PDB code: 2AJF. (c) The binding interface between HCoV-NL63 S-RBD (magenta) and ACE2 (green) based on PDB code: 3KBH.

**Table S1. Data collection and structure refinement statistics**

|  | SARS-CoV-2 S-RBD/ ACE2[W19/Y330] | SARS-CoV-2 S-RBD/ ACE2[W27/Y330] |
| --- | --- | --- |
| **Data collection** |  |  |
| Space group | P43212 | P43212 |
| Cell dimensions |  |  |
|  | 139.596, 139.596, 155.645 | 139.578, 139.578, 156.062 |
| α, β, γ (°) | 90.000, 90.000, 90.000 | 90.000, 90.000, 90.000 |
| Wavelength (Å) | 0.97915 | 0.97915 |
| Resolution (Å) | 50-2.70 (2.80-2.70) | 50-2.50 (2.59-2.50) |
| *R*_merge_ | 0.155 (1.297) | 0.126 (1.173) |
| *I* / sig*I* | 19.6 (2.0) | 26.0 (2.0) |
| Completeness (%) | 100.0(100.0) | 100.0 (100.0) |
| Redundancy | 12.3 (12.9) | 21.0 (19.9) |
|  |  |  |
| **Refinement** |  |  |
| Resolution (Å) | 25.92-2.70 | 26.28-2.49 |
| No. reflections | 42831 | 53980 |
| *R*_work_ / *R*_free_ | 0.1838/0.2242 | 0.1903/0.2289 |
| No. atoms |  |  |
| Protein | 6396 | 6395 |
| Water | 58 | 144 |
| *B*-factors |  |  |
| Protein | 57.34 | 53.88 |
| Water | 53.79 | 50.76 |
| R.m.s. deviations |  |  |
| Bond lengths (Å) | 0.009 | 0.010 |
| Bond angles (°) | 1.021 | 1.030 |
| Ramachandran plot (%)  Favored region  Allowed region  Outlier region | 95.80  3.82  0.38 | 96.82  3.05  0.13 |

In each case, a single crystal was used to collect the data.

Values in parentheses are for the highest-resolution shell.

**Table S2. IC50 values for the indicated ACE2 proteins (wild-type and mutants) determined with SARS-CoV-2 (wild-type, antibody-resistant and circulating variants) and SARS-CoV pseudoviruses (μg/ml)**

| **Pseudovirus** | **ACE2 protein** | | | |
| --- | --- | --- | --- | --- |
|  | **ACE2/WT** | **ACE2[W19/Y330]** | **ACE2[W27/Y330]** | **ACE2[W19/W27/Y330]** |
| **SARS-CoV-2** | 15.44 | 1.21 | 1.52 | 2.04 |
| **SARS-CoV** | 54.88 | 2.10 | 5.11 | 3.82 |
| **SARS-CoV-2 N439K** | 3.74 | 1.11 | 1.72 | 2.17 |
| **SARS-CoV-2 G446V** | 11.14 | 1.32 | 1.54 | 1.87 |
| **SARS-CoV-2 N450K** | 9.92 | 1.09 | 1.53 | 1.99 |
| **SARS-CoV-2 L452R** | 2.50 | 0.22 | 0.51 | 0.58 |
| **SARS-CoV-2 A475V** | 17.52 | 1.54 | 7.40 | 2.58 |
| **SARS-CoV-2 S477N** | 1.84 | 0.34 | 0.42 | 0.68 |
| **SARS-CoV-2 V483A** | 4.86 | 0.40 | 0.47 | 1.15 |
| **SARS-CoV-2 E484K** | 7.97 | 1.29 | 1.66 | 2.40 |
| **SARS-CoV-2 F490L** | 12.03 | 1.57 | 2.38 | 2.47 |
| **SARS-CoV-2 Y508H** | 3.69 | 0.54 | 0.65 | 1.10 |
| **SARS-CoV-2 B.1.351** | 2.98 | 1.11 | 1.42 | 2.20 |
| **SARS-CoV-2 B.1.617** | 4.74 | 0.47 | 0.90 | 1.21 |
|  | **ACE2/WT-Fc** | **ACE2[W19/Y330]-Fc** | **ACE2[W27/Y330]-Fc** | **ACE2[W19/W27/Y330]-Fc** |
| **SARS-CoV-2** | 1.44 | 0.09 | 0.25 | 0.53 |
| **SARS-CoV** | 4.17 | 0.34 | 1.04 | 0.49 |

**Table S3. The detailed hydrogen bond and van der Waals interactions between SARS-CoV-2 S-RBD and the ACE2 mutants**

| ACE2/WT ^a^ | SARS-CoV-2 S-RBD ^b^ | H-bond ^c^ |  | ACE2[W19/Y330] ^a^ | SARS-CoV-2 S-RBD ^b^ | H-bond ^c^ |  | ACE2[W27/Y330] ^a^ | SARS-CoV-2 S-RBD ^b^ | H-bond ^c^ |
| --- | --- | --- | --- | --- | --- | --- | --- | --- | --- | --- |
| S19 | 7 contacts (A475, G476) | 1 (A475) |  | W19 | 52 contacts (K458, Y473, Q474, A475, G476) |  |  | S19 |  |  |
| Q24 | 24 contacts (A475, G476, N487) | 1 (N487) |  | Q24 | 22 contacts (A475, G476, N487, Y489) | 2 (A475, N487) |  | Q24 | 25 contacts (A475, G476, S477, N487, Y489) | 1 (N487) |
| T27 | 15 contacts (F456, Y473, A475, Y489) |  |  | T27 | 13 contacts (F456, Y473, A475, Y489) |  |  | W27 | 35 contacts (F456, Y473, A475, Y489) |  |
| F28 | 7 contacts (Y489) |  |  | F28 | 9 contacts (Y489) |  |  | F28 | 9 contacts (Y489) |  |
| D30 | 10 contacts (K417, L455, F456) | 1 (K417) |  | D30 | 12 contacts (K417, L455, F456) | 1 (K417) |  | D30 | 13 contacts (K417, L455, F456) | 1 (K417) |
| K31 | 19 contacts (L455, F456, E484, Y489, F490, Q493) |  |  | K31 | 24 contacts (L455, F456, E484, Y489, F490, Q493) | 2 (Q493) |  | K31 | 26 contacts (L455, F456, E484, Y489, F490, Q493) | 1 (Q493) |
| H34 | 22 contacts (Y453, L455, Q493) |  |  | H34 | 15 contacts (K417, Y453, L455) |  |  | H34 | 14 contacts (Y453, L455, Q493) |  |
| E35 | 11 contacts (Q493) | 1 (Q493) |  | E35 | 1 contact (Q493) |  |  | E35 | 2 contacts (Q493) |  |
| E37 | 7 contacts (Y505) |  |  | E37 | 8 contacts (Y505) | 1 (Y505) |  | E37 | 8 contacts (Y505) | 1 (Y505) |
| D38 | 13 contacts (Y449, G496, Q498) | 1 (Y449) |  | D38 | 22 contacts (Y449, S494, Y495, G496, Q498) | 1 (Q498) |  | D38 | 17 contacts (Y449, G496, Q498) | 2 (Y449, Q498) |
| Y41 | 32 contacts (Q498, T500, N501) | 2 (T500, N501) |  | Y41 | 28 contacts (Q498, T500, N501) | 2 (T500) |  | Y41 | 23 contacts (Q498, T500, N501) | 3 (T500, N501) |
| Q42 | 20 contacts (G446, Y449, Q4 98) | 3 (G446, Y449, Q498) |  | Q42 | 14 contacts (G446, Y449, Q498) | 2 (G446, Q498) |  | Q42 | 15 contacts (G446, G447, Y449, Q498) | 2 (G446, Q498) |
| L45 | 6 contacts (Q498, T500) |  |  | L45 | 2 contacts (T500) |  |  | L45 | 2 contacts (T500) |  |
| L79 | 2 contacts (F486) |  |  | L79 | 3 contacts (F486) |  |  | L79 | 1 contact (F486) |  |
| M82 | 9 contacts (F486) |  |  | M82 | 12 contacts (F486) |  |  | M82 | 15 contacts (F486) |  |
| Y83 | 20 contacts (F486, N487, Y489) | 1 (N487) |  | Y83 | 19 contacts (F486, N487, Y489) | 1 (N487) |  | Y83 | 19 contacts (F486, N487, Y489) | 1 (N487) |
| N330 | 8 contacts (T500) |  |  | Y330 | 16 contacts (P499, T500) | 1(P499) |  | Y330 | 16 contacts (P499, T500) | 1(P499) |
| K353 | 48 contacts (G496, N501, G502, Y505) | 2 (G496, G502) |  | K353 | 60 contacts (G496, Q498, N501, G502, Y505) | 3 (G496, Q498, G502) |  | K353 | 59 contacts (G496, Q498, N501, G502, Y505) | 3 (G496, Q498, G502) |
| G354 | 11 contacts (G502, Y505) |  |  | G354 | 10 contacts (G502, Y505) |  |  | G354 | 11 contacts (G502, Y505) |  |
| D355 | 9 contacts (T500, G502) | 1 (T500) |  | D355 | 8 contacts (T500, G502) | 1(T500) |  | D355 | 8 contacts (T500, G502) | 1 (T500) |
| R357 | 3 contacts (T500) |  |  | R357 | 3 contacts (T500) |  |  | R357 | 3 contacts (T500) |  |
| R393 | 1 contact (Y505) |  |  | R393 | 2 contacts (Y505) |  |  | R393 | 3 contacts (Y505) |  |

^a^ ACE2 (ACE2/WT, ACE2[W19/Y330] and ACE2[W27/Y330]) residues that locate within a distance of 4.5 Å from the S-RBD ligand were analyzed by the contact program in CCP4 suite, selected and listed sequentially.

^b^ Numbers represent the number of atom-to-atom contacts contributed by each ACE2 residues listed in the left column. Those amino acids in S-RBD that locate within a distance of 4.5 Å from the corresponding ACE2 residue were listed in parentheses.

^c^ Numbers represent the number of hydrogen bonds contributed by the ACE2 residues listed in the left column. The S-RBD amino acids that form hydrogen bonds with the corresponding ACE2 residue were listed in parentheses. The distance cut-off is 3.5 Å.
